# Supplementary material for: Practical suggestions for harms reporting in exercise oncology: the Exercise Harms Reporting Method (ExHaRM)
Source: BMJ Open. 2022 Dec 8;12(12):e067998. doi: 10.1136/bmjopen-2022-067998 (PMC9743394; doi:10.1136/bmjopen-2022-067998)
Supplement: Supplementary data [file bmjopen-2022-067998supp001.pdf]

Practical suggestions for harms reporting in exercise oncology: the Exercise Harms Reporting Method (ExHaRM)

Rosalind R Spence, Carolina X Sandler, Tamara L Jones, Nicole M McDonald, Riley M Dunn, Sandra C Hayes

Supplemental material - Box 1: Key terms and definitions used in the Exercise Harms Reporting Method (ExHaRM)

**Glossary of terms** (in alphabetical order)	
**Active surveillance of harms**	Participants are asked via structured questionnaires or interviews about the occurrence of *adverse outcomes*, or laboratory or other diagnostic tests which are performed at prespecified time intervals.<br>In the most basic form, this may involve asking the participant or exercise professional about the occurrence of "any undesirable occurrences" at set-time points during the study.
**Adverse event**	*Adverse event* is the most common measurement tool in harms reporting across disciplines.<br><br>*Adverse events* are any harmful or undesirable medical occurrence that occurs during participation in an intervention, irrespective of whether there is a causal relationship with the intervention.<br>A medical occurrence may include (but is not limited to):<br>• an acute injury;<br>• exacerbation of a chronic or past injury;<br>• new treatment-related side effect or symptom;<br>• increase in severity of treatment-related side effect or symptom;<br>• study withdrawal;<br>• early termination of treatment;<br>• disease progression or death from disease during intervention period.[1]
**Adverse Outcome**	An *adverse outcome* is any undesirable physical, psychological, social and economic outcome, experience or event. *Adverse outcomes* are all undesirable occurrences, irrespective of whether there was a causal relationship between the outcome and the intervention.
**Adverse Outcomes of interest**	*Adverse outcomes* that are important to the consumer (people living with and beyond cancer), clinicians (medical and exercise professional) and decision makers.<br>*Adverse outcomes of interest* are determined based on previous evidence of the harms of specific exercise (e.g., mode, intensity) in a given population (i.e., oncology and haematology). These often include: the most clinically serious adverse outcomes, common adverse outcomes, and adverse outcomes of importance to stakeholders and patients.<br>Examples of *adverse outcomes of interest*:

Spence RR, *et al. BMJ Open* 2022; 12:e067998. doi: 10.1136/bmjopen-2022-067998

Practical suggestions for harms reporting in exercise oncology: the Exercise Harms Reporting Method (ExHaRM)

	• fracture rates in a population with pre-existing bone lesions (data collected via validated self-report questionnaires or extracted from medical records),<br>• newly diagnosed or exacerbated lymphoedema (this may be recorded by participants being prompted to report results of standard surveillance by their medical team or through study-specific testing, e.g., using bioelectrical impedance spectroscopy at pre-determined time points),<br>• issues with stomas or peripherally-inserted central catheters (data collected from medical records or prompted self-report at set time points).<br>For some *adverse outcomes of interest* it may be possible to collect data from the control and exercise groups, allowing comparison of risk.<br><br>Collecting *adverse outcomes of interest* should not exclude the active surveillance of other *adverse outcomes*, particularly given that what is important varies between individuals and situations.
**All-cause adverse outcomes**	*All-cause adverse outcomes* are all *adverse outcomes* identified in a trial, regardless of *causality* attribution.<br>*All-cause adverse outcomes* = non-exercise-related *adverse outcomes* + *exercise-related adverse outcomes.*
**Causality**	*Causality* describes the likelihood that an *adverse outcome* was caused by exercise, as judged by the exercise professional delivering the exercise intervention (or *Harms Panel*). *Causality* attribution is based on a range of factors, including the timing of the *adverse outcome* in relation to exercise versus other factors, the biological plausibility of the relationship and the expectedness of the *adverse outcome* (with regard to the nature, severity and frequency of the outcome) in the population being studied (i.e., is the adverse outcome an expected outcome of disease or treatment and therefore likely to occur even in the absence of exercise?).<br><br>**Categories:**<br>C = Certain: *adverse outcome* occurred within a plausible time relationship to completed exercise and cannot be explained by concurrent disease, treatment, or other drugs.<br><br>L = Likely: *adverse outcome* has a reasonable time sequence to completed exercise and unlikely to be attributed to concurrent disease, treatment, or other drugs. [sometimes termed “probable”, ExHaRM preferentially uses “likely” to reduce potential data errors due to confusing probable and possible]<br><br>P = Possible: *adverse outcome* has a reasonable time sequence to completed exercise but could also be explained by concurrent disease, treatment or other drugs.<br><br>U = Unlikely: *adverse outcome* has a temporal relationship to drug administration, treatment, or other medical event which makes a causal relationship with completed exercise improbable but not impossible, and other drugs or underlying disease provide a plausible explanation.

Practical suggestions for harms reporting in exercise oncology: the Exercise Harms Reporting Method (ExHaRM)

	NR = Not related: *adverse outcome* has no temporal relationship with exercise and/or *adverse outcome* has been attributed by other health professional to another cause (e.g., underlying disease or drug)<br><br>UC = Unclassified: This category should only be used when there is no way to categorise the *adverse outcome*. An exercise professional may use the term to identify an *adverse outcome* as requiring classification by the harms review panel, particularly when the exercise professional does not have appropriate scope or expertise to judge causality. A*dverse outcomes* recorded as unclassified by the exercise professional must be classified by the harms panel.<br><br>**Useful questions for exercise professional or Harms Panel to consider when attributing causality include:**<br>1. Is the *adverse outcome* a previously identified or a known response to exercise?<br>2. Has the *adverse outcome* occurred previously in this study (in this or another participant)?<br>3. Does the *adverse outcome* improve or stop when exercise is ceased?<br>4. Does the *adverse outcome* reoccur when exercise is re-commenced?<br>5. Is there a temporal relationship between the *adverse outcome* and exercise? Did exercise precede the *adverse outcome* and did the *adverse outcome* appear at an appropriate time interval after exercise (e.g., did the *adverse outcome* occur during exercise, or during a period post exercise that would mechanistically link the *adverse outcome* with the exercise. The post-exercise period may differ based on the specific *adverse outcome*. For example, a vasovagal episode is more likely to occur immediately post-exercise, whereas delayed-onset muscle soreness is more likely to occur 24-48 hours post-exercise)<br>6. Was the *adverse outcome* present at the beginning of the study (i.e., prior to commencing intervention / exercise)?<br>7. Can the participant's underlying clinical disease state (or comorbidities) explain the *adverse outcome*?<br>8. Are there any other potential causes for the *adverse outcome*?<br><br>(Questions adapted from "Oncology Clinical Trials: successful design, conduct and analysis",[2] and World Health Organisation–Uppsala Monitoring Centre Causality assessment system[3]).
**Common terminology criteria for adverse events (CTC-AE)**	The National Cancer Institute Common Terminology Criteria for Adverse Events (CTC-AE)[4] is a descriptive terminology which can be used for *harms* reporting.<br><br>A grading (severity) scale is provided for each adverse event term.<br><br>These criteria provide standardised language for describing the type and defining the severity of adverse events. The list of terms was developed to reflect *adverse events* within the context of medical treatment and therefore does not include a comprehensive list of terms relevant to exercise interventions. ExHarm uses the broad severity rating categories (i.e., grades 1 to 5) to grade the *severity* of *adverse outcomes* in the same way the CTC-AE grades *adverse events*, but primarily relies on MedDRA or other standardised language to describe the *adverse outcome*.
**Exercise-related adverse outcome**	An *adverse outcome* that is judged as having a reasonable causal relationship with participating in the exercise intervention.

Practical suggestions for harms reporting in exercise oncology: the Exercise Harms Reporting Method (ExHaRM)

	**Note:** 'reasonable causal relationship' means to convey, in general, that there is evidence or argument to suggest a causal relationship. "Reasonable causal relationship" includes the *causality* classifications of: "Possible", "Likely" and "Certain".<br><br>(Refer to *causality* term for full definitions of causality categories and recommended questions for determining attribution.)
**Harms panel**	The study-specific harms panel review the allocated causality of all *adverse outcomes*.<br><br>The composition of the panel will depend on the needs of the study. Most harms panels will include an exercise professional with substantial oncology experience, a medical oncologist, surgeon and/ or nurse, and senior research staff. It is preferable to include at least one member of the panel who has no other involvement in the study.
**Impact on intervention**	The impact of the *adverse outcome* describes how the participant's involvement in the intervention (i.e., exercise) is changed because of the *adverse outcome*.<br><br>Categories:<br>**M[1]:** modified exercise prescription (frequency, intensity, type, duration, volume)<br>**A[1]:** adjusted frequency of contact (e.g., increased or decreased frequency of contact with exercise professional),<br>**TI [2]:** temporary interruption from the intervention (contact and exercise),<br>**PW [2]:** permanent withdrawal from intervention (with or without withdrawal from the study),<br>**D:** Death<br><br>[1] Adjusted frequency of contact and modified exercise prescription may occur concurrently.<br>[2] Permanent withdrawal and temporary interruption may need to be coded or recoded retrospectively.
**Medical dictionary for Regulatory Activities (MedDRA®)[5]**	MedDRA® is the international medical terminology developed under the auspices of the International Council for Harmonisation of Technical Requirements for Pharmaceuticals for Human Use (ICH). It provides a standardised method to describe medical events.<br>Using standardised language allows for comparison between studies and aggregate reporting via meta-analyses.<br><br>MedDRA is searchable via a browser which is accessible via internet, mobile device or desktop (https://www.meddra.org/). Access requires a subscription which is free of charge for non-commercial / not for profit organisations.<br>MedDRA® trademark is registered by ICH.<br><br>A limitation of MedDRA is that it is medical terminology and does not capture some outcomes, such as abnormal responses to exercise. Study-specific language lists can be developed for use in instances that are not able to be described by MedDRA.

Practical suggestions for harms reporting in exercise oncology: the Exercise Harms Reporting Method (ExHaRM)

**Non-systematic surveillance of harms**	Collection of *adverse outcomes* that does not use a systematic approach (i.e., researchers do not ask about specific *adverse outcomes* or apply standardised testing). This approach usually involves asking participants to report the presence of *adverse outcomes* they have noticed (this can be through active or passive surveillance).
**Passive surveillance of harms**	Participants are not specifically asked about or tested for the occurrence of *adverse outcome*. Rather, *adverse outcomes* are identified based on participant or exercise professional reports made on their own initiative. This is also called spontaneous reporting.
**Persistent adverse outcome**	A *persistent adverse outcome* is an *adverse outcome* that extends continuously, without resolution.<br>The *adverse outcome* is reported only once unless the grade changes. If the grade becomes more or less severe the *adverse outcome* is reported again, as a new *adverse outcome* with the new grade.
**Recall period**	The period between each occasion of *adverse outcome* reporting (by participant or clinician).<br>E.g., If the exercise professional asks the participant to report any *adverse outcomes* that have occurred since the previous session that was 7 days ago, then the *recall period* would be 7 days.
**Recurrent adverse outcome**	A *recurrent adverse outcome* is an *adverse outcome* that occurs and resolves.<br>An *adverse outcome* that resolves and then recurs should be reported at each recurrence.
**Serious adverse event**	The term *Serious adverse event* has not been used in in ExHaRM. We acknowledge the term here as it is often a reporting requirement of ethics or institutional boards.<br><br>*Serious adverse events* are *adverse events* that meet a specific definition, separate to severity. The term is primarily used in defining regulatory reporting obligations for clinical trials. In ExHaRM *adverse events* are captured by the umbrella term *adverse outcome. Adverse outcomes* are divided by grade into mild-moderate (grade 1-2) and severe (≥ grade 3), rather than being defined as serious and non-serious.<br><br>*Serious adverse events*[6] are any <u>exercise-related</u> *adverse events* (i.e., "undesirable medical occurrences") occurring at any dose of an intervention that results in ANY of the following outcomes:<br>1) Death.<br>2) A life-threatening experience.<br>3) Inpatient hospitalization or prolongation of existing hospitalization (for >24 hours).<br>4) A persistent or significant incapacity or substantial disruption of the ability to conduct normal life functions<br>5) A congenital anomaly/birth defect.<br>6) Important Medical Events that may not result in death, be life threatening, or require hospitalization may be considered a serious adverse experience when, based upon medical judgment, they may jeopardize the patient or subject and may require medical or surgical intervention to prevent one of the outcomes listed in this definition

Practical suggestions for harms reporting in exercise oncology: the Exercise Harms Reporting Method (ExHaRM)

	Reporting requirements are usually determined by ethics or institutional boards and in most cases there are specific reporting requirements for *serious adverse events*. Given clinical and legal implications of these events, it is recommended that all exercise interventions and programs have a process requiring exercise professionals to report these *serious adverse events* to senior staff. Further, it is recommended that any *adverse outcomes* that meet the above definition should be reported to supervisors/ senior staff for review, regardless of whether the exercise professional judged the event to be exercise-related. It is recommended that *serious adverse events* (or *adverse outcomes* where *causality* could not be confidently assessed, but that otherwise met any serious adverse event criteria) are reported to the harms panel within 48 hours for immediate review.
**Severity / Grade**	*Adverse outcomes* are given a severity rating based on a grading scale of 1-5 (as per CTC-AE).<br><br>Severity may also be grouped into categories of mild-moderate (grade 1-2 *adverse outcomes*) or severe (grade 3-5 *adverse outcomes*).<br>Note: A severe *adverse outcome*, is NOT the same as *serious adverse event* which is defined separately (see "*serious adverse events*")
**Grade 1**	Mild; asymptomatic or mild symptoms; clinical or diagnostic observations only; medical treatment not indicated.<br><br>*ExHaRM application: "no/minimal disruption to activities of daily living"*
**Grade 2**	Moderate; minimal, local or noninvasive medical treatment indicated; limiting age-appropriate instrumental activities of daily living.<br>[Instrumental activities of daily living refer to activities such as preparing meals, shopping for groceries, using the telephone, managing money, etc.]<br><br>*ExHaRM application: "some disruption to instrumental activities of daily living"*
**Grade 3**	Severe or medically significant but not immediately life-threatening; hospitalization or prolongation of hospitalization indicated; disabling; substantially limiting self-care activities of daily living.<br>[Self-care activities of daily living refer to activities including bathing, dressing and undressing, feeding self, using the toilet, taking medications.]<br><br>*ExHaRM application: "complete disruption to self-care activities of daily living"*
**Grade 4**	Life-threatening consequences; urgent medical treatment indicated.
**Grade 5**	Death related to *adverse outcome*.
**Systematic surveillance of harms**	Asking every participant (at defined time points) about specific *adverse outcomes or* performing specific laboratory or other diagnostic tests.<br>Systematic surveillance is, by definition, one approach to active surveillance of harms.

Practical suggestions for harms reporting in exercise oncology: the Exercise Harms Reporting Method (ExHaRM)

	Systematic methods, such as querying participants using a comprehensive checklist or standardized laboratory tests, are more likely than non-systematic methods to identify all occurrences of an *adverse outcome*.
**Type of adverse outcome**	Relevant categories of *Type of adverse outcome* may depend on the type of intervention and any *harms of interest* collected.<br><br>*Example list and reporting codes*<br>**Ex:** normal response to exercise (e.g., DOMS),<br>**AbEx:** abnormal response to exercise,<br>**SE:** expected disease- or treatment-related side effect,<br>**SE+:** exacerbation of disease- or treatment-related side effect,<br>**In:** injury,<br>**O:** other (describe)

References

1. INTERNATIONAL COUNCIL FOR HARMONISATION OF TECHNICAL REQUIREMENTS FOR PHARMACEUTICALS FOR HUMAN USE (ICH), *Indexed ICH GCP guidelines with integrated addendum E6(R2), Step 4*. First edition, ed. D.R. Hutchinson. 2016, Chobham, Surrey: International council for harmonisation.
2. Kelly, W. and S. Halabi, *Oncology Clinical Trials: Successful Design, Conduct, and Analysis*. 2nd ed. 2018.
3. World Health Organization (WHO)-Uppsala Monitoring Centre *The use of the WHO-UMC system for standardized case causality assessment*.
4. National Cancer Institute (U.S.), *Common terminology criteria for adverse events (CTCAE) - version 5.0*. NIH publication. 2017, Bethesda, Md.: U.S. Dept. of Health and Human Services, National Institutes of Health, National Cancer Institute.
5. International Council for Harmonisation of Technical Requirements for Pharmaceuticals for Human Use, *MedDRA® Medical Dictionary for Regulatory Activities terminology. Retrieved from https://www.meddra.org/*. 2020.
6. National Institutes of Health (NIH), *NCI Guidelines For Investigators: ADVERSE EVENT REPORTING REQUIREMENTS*, U.S. Dept. of Health & Human Services, Editor. 2013.
