# Supplementary material for: Practical suggestions for harms reporting in exercise oncology: the Exercise Harms Reporting Method (ExHaRM)
Source: BMJ Open. 2022 Dec 8;12(12):e067998. doi: 10.1136/bmjopen-2022-067998 (PMC9743394; doi:10.1136/bmjopen-2022-067998)
Supplement: Author's manuscript [file bmjopen-2022-067998.draft_revisions.pdf]

BMJ Open

BMJ Open is committed to open peer review. As part of this commitment we make the peer review history of every article we publish publicly available.

When an article is published we post the peer reviewers' comments and the authors' responses online. We also post the versions of the paper that were used during peer review. These are the versions that the peer review comments apply to.

The versions of the paper that follow are the versions that were submitted during the peer review process. They are not the versions of record or the final published versions. They should not be cited or distributed as the published version of this manuscript.

BMJ Open is an open access journal and the full, final, typeset and author-corrected version of record of the manuscript is available on our site with no access controls, subscription charges or pay-per-view fees (http://bmjopen.bmj.com).

If you have any questions on BMJ Open's open peer review process please email info.bmjopen@bmj.com

BMJ Open

First do no harm: practical suggestions for harms reporting in Exercise Oncology

Journal:	*BMJ Open*
Manuscript ID	bmjopen-2022-067998.R1
Article Type:	Communication
Date Submitted by the Author:	10-Oct-2022
Complete List of Authors:	Spence, Rosalind; Griffith University, Menzies Institute of Health Queensland; Griffith University, Improving Health Outcomes for People (ihop) research group, School of Health Sciences and Social Work<br>Sandler, Carolina; Griffith University, Improving Health Outcomes for People (ihop) research group, School of Health Sciences and Social Work; Western Sydney University, Sport and Exercise Science, School of Health Science<br>Jones, Tamara; Griffith University, Improving Health Outcomes for People (ihop) research group, School of Health Sciences and Social Work; University of Melbourne, Melbourne School of Psychological Sciences, Faculty of Medicine, Dentistry, and Health Sciences<br>McDonald, Nicole; Griffith University, Menzies Health Institute Queensland; Griffith University, Improving Health Outcomes for People (ihop) research group, School of Health Sciences and Social Work<br>Dunn, Riley; Griffith University, Menzies Health Institute Queensland; Griffith University, Improving Health Outcomes for People (ihop) research group, School of Health Sciences and Social Work<br>Hayes, Sandra; Griffith University, Menzies Health Institute Queensland; Griffith University, Improving Health Outcomes for People (ihop) research group, School of Health Sciences and Social Work
**Primary Subject Heading**:	Sports and exercise medicine
Secondary Subject Heading:	Oncology
Keywords:	SPORTS MEDICINE, PUBLIC HEALTH, ONCOLOGY

SCHOLARONE™ Manuscripts

I, the Submitting Author has the right to grant and does grant on behalf of all authors of the Work (as defined in the below author licence), an exclusive licence and/or a non-exclusive licence for contributions from authors who are: i) UK Crown employees; ii) where BMJ has agreed a CC-BY licence shall apply, and/or iii) in accordance with the terms applicable for US Federal Government officers or employees acting as part of their official duties; on a worldwide, perpetual, irrevocable, royalty-free basis to BMJ Publishing Group Ltd ("BMJ") its licensees and where the relevant Journal is co-owned by BMJ to the co-owners of the Journal, to publish the Work in BMJ Open and any other BMJ products and to exploit all rights, as set out in our licence.

The Submitting Author accepts and understands that any supply made under these terms is made by BMJ to the Submitting Author unless you are acting as an employee on behalf of your employer or a postgraduate student of an affiliated institution which is paying any applicable article publishing charge ("APC") for Open Access articles. Where the Submitting Author wishes to make the Work available on an Open Access basis (and intends to pay the relevant APC), the terms of reuse of such Open Access shall be governed by a Creative Commons licence – details of these licences and which Creative Commons licence will apply to this Work are set out in our licence referred to above.

Other than as permitted in any relevant BMJ Author's Self Archiving Policies, I confirm this Work has not been accepted for publication elsewhere, is not being considered for publication elsewhere and does not duplicate material already published. I confirm all authors consent to publication of this Work and authorise the granting of this licence.

For peer review only

First do no harm: practical suggestions for harms reporting in Exercise Oncology – ExHaRM

Rosalind R Spence[1, 2], Carolina X Sandler[2, 3], Tamara L Jones[2, 4], Nicole M McDonald[1, 2], Riley M Dunn[1, 2], Sandra C Hayes[1, 2]

[1] Menzies Health Institute Queensland, Griffith University, Brisbane, Australia

[2] Improving Health Outcomes for People (ihop) research group, School of Health Sciences and Social Work, Griffith University, Brisbane, Australia

[3] Sport and Exercise Science, School of Health Science, Western Sydney University, Sydney, NSW, Australia.

[4] Melbourne School of Psychological Sciences, Faculty of Medicine, Dentistry, and Health Sciences, University of Melbourne

Word Count: 1657
Tables: 1
Figures: 1

Corresponding author: Rosalind R Spence
Email: r.spence@griffith.edu.au
Postal: Menzies Health Institute Queensland, Griffith 43 University, Brisbane, Australia.
Postal address: Griffith University Nathan Campus, 170 Kessels Road, 44 NATHAN, QLD 4111

Keywords: cancer; adverse events; clinical trial reporting; safety; harms

For peer review only

Abstract

The volume of high-quality evidence supporting exercise as beneficial to cancer survivors has grown exponentially; however, the potential harms of exercise remain understudied. Consequently, the trade-off between desirable and undesirable outcomes of engaging in exercise remains unclear to clinicians and people with cancer. Practical guidance on collecting and reporting harms in exercise oncology is lacking. We present a harms reporting protocol developed and refined through exercise oncology trials since 2015.

Development of the Exercise Harms Reporting Method (*ExHaRM)* was informed by national and international guidelines for harms reporting in clinical trials involving therapeutic goods or medical devices, with adaptations to enhance applicability to exercise. The protocol has been adjusted via an iterative process of implementation and adjustment through use in multiple exercise oncology trials involving varied cancer diagnoses (types: breast, brain, gynaecological; stages at diagnosis I-IV; primary/recurrent), and heterogeneous exercise intervention characteristics (face-to-face/telehealth delivery; supervised/unsupervised exercise). It has also involved the development of terms (such as, adverse outcomes, which capture all undesirable physical, psychological, social and economic outcomes) that facilitate the harms assessment process in exercise.

ExHaRM involves: Step 1: Monitor occurrence of adverse outcomes through systematic and non-systematic surveillance; Step 2: Assess and record adverse outcomes, including severity, causality, impact on intervention and type; Step 3: Review of causality by harms panel (and revise as necessary); and Step 4: Analyse and report frequencies, rates and clinically meaningful details of all-cause and exercise-related adverse outcomes.

ExHaRM provides guidance to improve the quality of harms assessment and reporting immediately, while concurrently providing a framework for future refinement. Future directions include, but are not limited to, standardising exercise-specific nomenclature and methods of assessing causality.

For peer review only

Introduction

Since publication of the first exercise oncology prescription guidelines over a decade ago, [1, 2] the volume and quality of evidence supporting the benefits of exercise following a cancer diagnosis has increased exponentially.[3, 4] The current model of evidence-based medicine is founded on the principle that expected benefits, as well as potential harms, alongside patient values and preferences, drive clinician recommendations and patient decisions.[5] Unfortunately, the harms of exercise remain understudied and underreported in exercise trials (missing or incompletely reported in 51-75% of studies),[6, 7] and specifically, within exercise oncology (not reported in 34-47% of studies).[8, 9] Harms are "the totality of possible adverse consequences of an intervention or therapy; they are the direct opposite of benefits, against which they must be compared."[10] The absence of data regarding the harms of exercise significantly compromises the ability of clinicians, patients and exercise professionals to evaluate the trade-off between desirable and undesirable consequences.[5] There is insufficient evidence for (i) clinicians to judge the safety profile of exercise as a potential adjuvant therapy, (ii) patients to make informed decisions about potential consequences of exercise participation, and (iii) exercise professionals to determine the appropriateness of exercise (i.e., contraindications, or dosage and prescription considerations).

Despite a growing recognition of the importance of accurate and comprehensive reporting of the harms of exercise,[7] practical guidance on how to manage the collection and reporting process in exercise oncology interventions is missing.[8, 9] Well-accepted standards and guidelines for harms reporting in pharmacological cancer clinical trials exist.[11] However, the direct application of these frameworks to exercise trials, without modification, is inappropriate.[12] Pharmacological harms are assessed via adverse events (i.e., "any undesirable medical occurrence" [13]). However, the collection of only adverse events fails to capture the breadth of potential negative consequences that may occur due to behavioural interventions, including exercise.[12] Harms reporting in exercise interventions is further complicated by the need to distinguish harms associated with medical treatment from those associated with exercise. Below we share our approach to exercise harms reporting, with the aim of encouraging more comprehensive reporting of harms data and providing a platform and framework from which future studies can build.

Exercise Harms Reporting Method

For peer review only - http://bmjopen.bmj.com/site/about/guidelines.xhtml

The **Ex**ercise **Ha**rms **R**eporting **M**ethod (ExHaRM) is a protocol for the monitoring and reporting of exercise-related harms. It was developed based on guidelines for harms reporting in clinical trials involving therapeutic goods or medical devices (specifically, Good Clinical Practice guidelines;[13] National Cancer Institute Common terminology criteria for AEs [CTC-AE][11]), as well as international,[14] Australian and local institutional reporting requirements,[15] and the expert opinions of trial investigators (including exercise oncology researchers, accredited exercise physiologists, cancer nurses, breast surgeons, gynaecological oncologists and medical oncologists). The protocol has been refined through an iterative process of implementation and adjustment since it was first developed in 2015. This includes exercise oncology trials[16-19], involving samples with breast, brain and gynaecological cancer, different stages of disease at diagnosis (stage I-IV), primary and recurrent diagnoses, and the presence of multiple comorbidities. Further, ExHaRM has been used in studies that involve mixed-mode exercise (aerobic and resistance training) delivered during and following treatment, via various modes of delivery (face-to-face supervision ranging from 2 sessions/week to 1 session/month, in addition to trials delivered via tele-health), and a range of settings (e.g., exercise completed at participants' homes, community fitness centres, and university exercise clinics).

ExHaRM defines the harms of exercise as all undesirable physical, psychological, economic or social consequences (covering incidences, experiences, occurrences) that are related to an individual's participation in exercise. Given assessment of **adverse events** does not fully capture harms of exercise, we therefore propose the use of the term **adverse outcomes** to assess undesirable physical, psychological, social and economic outcomes, experiences or events, irrespective of whether they have a causal relationship with exercise. ExHaRM involves four core steps (summarised in Figure 1 and explained in Table 1) to capture, classify, analyse and report all-cause and exercise-related adverse outcomes. A downloadable and interactive version of ExHaRM is available at www.improvinghealth.com.au/projects/exharm). Key terms, including their definitions and related information (e.g., prompts or questions to ensure accurate data collection, and coding recommendations to aid efficient recording) are denoted by italics throughout the method and can be found at the end of Table 1.

For peer review only - http://bmjopen.bmj.com/site/about/guidelines.xhtml

Table 1: Overview of the Exercise Harms Reporting Method (ExHaRM), including key terms and definitions

The **Ex**ercise **Ha**rms **R**eporting **M**ethod (ExHaRM)
The purpose of this protocol is to guide the collection and reporting of informative data on the *harms of exercise*.
Note: Italicised terms are defined at the end of the table.
**1) Monitor & Identify**<br>Systematically monitor and identify *adverse outcomes*:<br>• Actively prompt participants to report *adverse outcomes*. Use standardised language and standardised *recall periods*. For example, the exercise professional asks the participant at every contact: "Since our last session have you experienced any harmful or undesirable outcomes, whether or not you think they were caused by exercise? For example, have you had an injury or worsening of an old injury, or any unusual or worsening treatment-related symptom?" (i.e., *active surveillance*)<br>• Train exercise professionals to actively observe for *adverse outcomes* that may occur during supervised exercise, and to probe during discussions if a participant reports something that might be an *adverse outcome* (i.e., *passive surveillance* / spontaneous reporting of *adverse outcomes*).<br>• Consider generating a study-specific list of *adverse outcomes of interest* that are systematically recorded via a checklist or standardised test at fixed time points (*systematic surveillance*), in addition to the broad question recommended above (*non-systematic surveillance*).<br><br>**Respond**<br>Whilst not part of the harms-reporting process, the exercise professional has a duty of care to the participant to identify, consider and manage all *adverse outcomes* and depending on the nature of the *adverse outcome* (with particular attention required for *adverse events*) may have external reporting requirements:

- Provide first aid and management of the *adverse outcome*: Respond to immediate needs of the participant through administration of first aid, cessation or modification of exercise, referral to other health professional, and/or other responses as required.
- Complete tasks as per exercise professional duties and scope of practice: Consider whether *adverse outcome* requires an absence from exercise (i.e., is it a contraindication?) and/or modification of exercise parameters (e.g., volume, intensity, or type).
- Report as per requirements of employer, institution, funding and / or ethics committees.

Note: The definitions (e.g., of *harms*, *adverse outcomes*, and *severity*) in this protocol are those determined to be most appropriate to the evaluation of exercise-related harms. Regulatory committees (e.g., a human research ethics committee) typically require the reporting of "*serious adverse events*" only, which will be captured within the monitoring and reporting of *adverse outcomes* as outlined in this protocol.

2) Assess & Record

Assess and record the *adverse outcome* and associated classifications of *severity, causality, impact of adverse outcome on the intervention,* and *type of adverse outcome,* including sufficient detail to justify the assigned category. Also record detail of the context and sequalae of the *adverse outcome*; for example, where and when the *adverse outcome* occurred, what clinical action was taken, the duration of the *adverse outcome* and any subsequent treatment.

Severity (Grade 1-5): based primarily on the resultant medical treatment and the impact of the *adverse outcome* on activities of daily living.

Causality: based on the timing of the *adverse outcome* in relation to exercise and plausibility of relationship with exercise or other factors (see useful questions to consider in Table 1 under "*Causality*"). Include both the participant's opinion and exercise professional's judgement of *causality*.

For peer review only - http://bmjopen.bmj.com/site/about/guidelines.xhtml

Impact on intervention: based on changes to a participant's involvement in the exercise intervention caused by the *adverse outcome*. This may involve modifications (e.g., modified exercise prescription, missed exercise sessions), or absences from the intervention (either temporary or permanent).

Type of adverse outcome: the purpose of categorising *adverse outcome* by type is to create groupings for simplified summary based on predetermined categories (e.g., abnormal response to exercise, injury, exacerbation of treatment-related side effect).

Note: Care should be taken to differentiate between a *persistent* and *recurrent adverse outcome*, as *persistent adverse outcomes* need only be reported once, whereas *recurrent adverse outcomes* should be reported at each recurrence.

3) Review by *Harms Panel* and potential revision of causality

The attribution of *causality* (i.e., determining which *adverse outcomes* are causally related to exercise) is essential to the evaluation of exercise-related harms. *Causality* attribution requires knowledge of the mechanisms by which an intervention may cause harm, as well as the expected responses to an intervention (i.e., scope of exercise professional). Concurrently, an understanding of what is common and expected in the population (both disease- and treatment-related) is also required and is likely most appropriately provided by the medical team. Therefore, while it is appropriate for exercise professionals to make the initial judgement of causality, ideally *adverse outcomes* should be reviewed by a panel with additional expertise, and preferably at least one individual who is not directly involved in the study. All *adverse outcomes* are reported to the study *harms panel* on a pre-determined schedule. This may be frequently (e.g., monthly) or at a single point prior to data analysis.

The *harms panel* review all *adverse outcomes* with special attention to:

- unusual or unanticipated *adverse outcomes* (as judged by panel);
- discordant opinions of causality recorded between participant and exercise professional;
- *adverse outcomes* ≥ grade 3.
- *adverse outcomes* that the exercise professional has highlighted for review

Discrepancies in *causality* attribution between exercise professional and any member of the harms panel should be discussed, and the consensus reached by the panel be recorded as the final *causality* decision.

The *harms panel* may also determine that a reported *adverse outcome* does not meet the definition of an *adverse outcome* (e.g., the recorded event might not be judged by the panel as being undesirable). In this situation the *adverse outcome* would be removed from the dataset and would not be reported.

4) Analyse & Report

4.1 Data preparation and analysis

- Any *adverse outcome* with a *causality* of certain, likely, or possible is categorised as an *exercise-related adverse outcome*. The term *"all-cause adverse outcomes"* is then used to refer to *adverse outcome* of any *causality* (i.e., all *adverse outcomes*: those deemed exercise related, as well as those not attributed to exercise).
- Re-code *adverse outcomes* with standardised language: each *adverse outcome* identified in the study should be described using standardised language (e.g., *Medical dictionary for Regulatory Activities* [MedDRA®][20] preferred terms) and summarised (e.g., within system organ class or appropriate groupings). For example: a report of "stiff and sore shoulders" as an *adverse outcome* in the clinical notes could be coded as "arthralgia" using *MedDRA terminology* and listed under the category of "Musculoskeletal and Connective Tissue Disorders", along with other conditions, such as arthritis.
- Group all-cause *adverse outcomes* and *exercise-related adverse outcomes* by each category of *Type of Adverse outcome* and *Impact on intervention*.

4.2 Reporting

All-cause and *Exercise-related adverse outcomes:*

For peer review only

- Report the number of *all-cause adverse outcomes* and *exercise-related adverse outcomes*, including the number (and proportion) of *all-cause adverse outcomes* and *exercise-related adverse outcomes* categorised by *severity* as mild-moderate (grades 1 and 2) and severe (grades 3-5).
- For these harms outcomes (i.e., number of all-cause *adverse outcomes* and *exercise-related adverse outcomes* by severity categories) it is helpful to also report the number of participants (and proportion of all participants) reporting an *adverse outcome* at least once.
- The number of *adverse outcomes* (and percentage) for each MedDRA code (e.g., arthralgia) and system organ class (e.g., musculoskeletal and connective tissue disorders), or other standardised language groupings, should be reported for all-cause *adverse outcomes* and for *exercise-related adverse outcomes*.

Exercise-related adverse outcomes only*:*

- The number (and proportion) of *exercise-related adverse outcomes* in each category of '*impact on intervention*' and '*type of adverse outcome*' should be reported.
- Highlight any unique or key *exercise-related adverse outcomes* relevant to the context of the study, or of clinical relevance (e.g., lymphoedema, dislodged peripherally-inserted central catheter, bone fractures).
- Consider reporting *exercise-related adverse outcomes* as a rate per person per week of intervention, in conjunction with the average weekly minutes of exercise that were completed. If rate is not reported, then ensure sufficient intervention and trial details are included to allow subsequent studies to compare results.

- *Adverse outcomes* that occur during exercise testing (i.e., objective data collection) should be reported separately to those that occur during the intervention.

Glossary of terms (in alphabetical order)

For peer review only

**Active surveillance of harms**	Participants are asked via structured questionnaires or interviews about the occurrence of *adverse outcomes*, or laboratory or other diagnostic tests which are performed at prespecified time intervals.<br>In the most basic form, this may involve asking the participant or exercise professional about the occurrence of "any undesirable occurrences" at set-time points during the study.
**Adverse event**	*Adverse event* is the most common measurement tool in harms reporting across disciplines.<br><br>*Adverse events* are any harmful or undesirable medical occurrence that occurs during participation in an intervention, irrespective of whether there is a causal relationship with the intervention.<br>A medical occurrence may include (but is not limited to):<br>• an acute injury;<br>• exacerbation of a chronic or past injury;<br>• new treatment-related side effect or symptom;<br>• increase in severity of treatment-related side effect or symptom;<br>• study withdrawal;<br>• early termination of treatment;<br>• disease progression or death from disease during intervention period.[13]
**Adverse Outcome**	An *adverse outcome* is any undesirable physical, psychological, social and economic outcome, experience or event.<br>*Adverse outcomes* are all undesirable occurrences, irrespective of whether there was a causal relationship between the outcome and the intervention.
**Adverse Outcomes of interest**	*Adverse outcomes* that are important to the consumer (people living with and beyond cancer), clinicians (medical and exercise professional) and decision makers.<br>*Adverse outcomes of interest* are determined based on previous evidence of the harms of specific exercise (e.g., mode, intensity) in a given population (i.e., oncology and haematology). These often include: the most clinically serious adverse outcomes, common adverse outcomes, and adverse outcomes of importance to stakeholders and patients.<br>Examples of *adverse outcomes of interest*:<br>• fracture rates in a population with pre-existing bone lesions (data collected via validated self-report questionnaires or extracted from medical records),<br>• newly diagnosed or exacerbated lymphoedema (this may be recorded by participants being prompted to report results of standard surveillance by their medical team or through study-specific testing, e.g., using bioelectrical impedance spectroscopy at pre-determined time points),<br>• issues with stomas or peripherally-inserted central catheters (data collected from medical records or prompted self-report at set time points).<br>For some *adverse outcomes of interest* it may be possible to collect data from the control and exercise groups, allowing comparison of risk.

For peer review only - http://bmjopen.bmj.com/site/about/guidelines.xhtml

	Collecting *adverse outcomes of interest* should not exclude the active surveillance of other *adverse outcomes*, particularly given that what is important varies between individuals and situations.
**All-cause adverse outcomes**	*All-cause adverse outcomes* are all *adverse outcomes* identified in a trial, regardless of *causality* attribution.<br>*All-cause adverse outcomes* = non-exercise-related *adverse outcomes* + *exercise-related adverse outcomes.*
**Causality**	*Causality* describes the likelihood that an *adverse outcome* was caused by exercise, as judged by the exercise professional delivering the exercise intervention (or *Harms Panel*). *Causality* attribution is based on a range of factors, including the timing of the *adverse outcome* in relation to exercise versus other factors, the biological plausibility of the relationship and the expectedness of the *adverse outcome* (with regard to the nature, severity and frequency of the outcome) in the population being studied (i.e., is the adverse outcome an expected outcome of disease or treatment and therefore likely to occur even in the absence of exercise?).<br><br>**Categories:**<br>C = Certain: *adverse outcome* occurred within a plausible time relationship to completed exercise and cannot be explained by concurrent disease, treatment, or other drugs.<br><br>L = Likely: *adverse outcome* has a reasonable time sequence to completed exercise and unlikely to be attributed to concurrent disease, treatment, or other drugs. [sometimes termed "probable", ExHaRM preferentially uses "likely" to reduce potential data errors due to confusing probable and possible]<br><br>P = Possible: *adverse outcome* has a reasonable time sequence to completed exercise but could also be explained by concurrent disease, treatment or other drugs.<br><br>U = Unlikely: *adverse outcome* has a temporal relationship to drug administration, treatment, or other medical event which makes a causal relationship with completed exercise improbable but not impossible, and other drugs or underlying disease provide a plausible explanation.<br><br>NR = Not related: *adverse outcome* has no temporal relationship with exercise and/or *adverse outcome* has been attributed by other health professional to another cause (e.g., underlying disease or drug)<br><br>UC = Unclassified: This category should only be used when there is no way to categorise the *adverse outcome*. An exercise professional may use the term to identify an *adverse outcome* as requiring classification by the harms review panel, particularly when the exercise professional does not have appropriate scope or expertise to

For peer review only

	judge causality. A*dverse outcomes* recorded as unclassified by the exercise professional must be classified by the harms panel.<br><br>**Useful questions for exercise professional or Harms Panel to consider when attributing causality include:**<br>1. Is the *adverse outcome* a previously identified or a known response to exercise?<br>2. Has the *adverse outcome* occurred previously in this study (in this or another participant)?<br>3. Does the *adverse outcome* improve or stop when exercise is ceased?<br>4. Does the *adverse outcome* reoccur when exercise is re-commenced?<br>5. Is there a temporal relationship between the *adverse outcome* and exercise? Did exercise precede the *adverse outcome* and did the *adverse outcome* appear at an appropriate time interval after exercise (e.g., did the *adverse outcome* occur during exercise, or during a period post exercise that would mechanistically link the *adverse outcome* with the exercise. The post-exercise period may differ based on the specific *adverse outcome*. For example, a vasovagal episode is more likely to occur immediately post-exercise, whereas delayed-onset muscle soreness is more likely to occur 24-48 hours post-exercise)<br>6. Was the *adverse outcome* present at the beginning of the study (i.e., prior to commencing intervention / exercise)?<br>7. Can the participant's underlying clinical disease state (or comorbidities) explain the *adverse outcome*?<br>8. Are there any other potential causes for the *adverse outcome*?<br><br>(Questions adapted from "Oncology Clinical Trials: successful design, conduct and analysis",[21] and World Health Organisation–Uppsala Monitoring Centre Causality assessment system[22]).
**Common terminology criteria for adverse events (CTC-AE)**	The National Cancer Institute Common Terminology Criteria for Adverse Events (CTC-AE) [11] is a descriptive terminology which can be used for *harms* reporting.<br><br>A grading (severity) scale is provided for each adverse event term.<br><br>These criteria provide standardised language for describing the type and defining the severity of adverse events. The list of terms was developed to reflect *adverse events* within the context of medical treatment and therefore does not include a comprehensive list of terms relevant to exercise interventions. ExHarm uses the broad severity rating categories (i.e., grades 1 to 5) to grade the *severity* of *adverse outcomes* in the same way the CTC-AE grades *adverse events*, but primarily relies on MedDRA or other standardised language to describe the *adverse outcome*.
**Exercise-related adverse outcome**	An *adverse outcome* that is judged as having a reasonable causal relationship with participating in the exercise intervention.<br><br>**Note:** 'reasonable causal relationship' means to convey, in general, that there is evidence or argument to suggest a causal relationship. "Reasonable causal relationship" includes the *causality* classifications of: "Possible", "Likely" and "Certain".

For peer review only

	(Refer to *causality* term for full definitions of causality categories and recommended questions for determining attribution.)
**Harms panel**	The study-specific harms panel review the allocated causality of all *adverse outcomes*.<br><br>The composition of the panel will depend on the needs of the study. Most harms panels will include an exercise professional with substantial oncology experience, a medical oncologist, surgeon and/ or nurse, and senior research staff. It is preferable to include at least one member of the panel who has no other involvement in the study.
**Impact on intervention**	The impact of the *adverse outcome* describes how the participant's involvement in the intervention (i.e., exercise) is changed because of the *adverse outcome*.<br><br>Categories:<br>**M[1]:** modified exercise prescription (frequency, intensity, type, duration, volume)<br>**A[1]:** adjusted frequency of contact (e.g., increased or decreased frequency of contact with exercise professional),<br>**TI [2]:** temporary interruption from the intervention (contact and exercise),<br>**PW [2]:** permanent withdrawal from intervention (with or without withdrawal from the study),<br>**D:** Death<br><br>[1] Adjusted frequency of contact and modified exercise prescription may occur concurrently.<br>[2] Permanent withdrawal and temporary interruption may need to be coded or recoded retrospectively.
**Medical dictionary for Regulatory Activities (MedDRA®)[20]**	MedDRA® is the international medical terminology developed under the auspices of the International Council for Harmonisation of Technical Requirements for Pharmaceuticals for Human Use (ICH). It provides a standardised method to describe medical events.<br>Using standardised language allows for comparison between studies and aggregate reporting via meta-analyses.<br><br>MedDRA is searchable via a browser which is accessible via internet, mobile device or desktop (https://www.meddra.org/). Access requires a subscription which is free of charge for non-commercial / not for profit organisations.<br>MedDRA® trademark is registered by ICH.<br><br>A limitation of MedDRA is that it is medical terminology and does not capture some outcomes, such as abnormal responses to exercise. Study-specific language lists can be developed for use in instances that are not able to be described by MedDRA.

For peer review only - http://bmjopen.bmj.com/site/about/guidelines.xhtml

**Non-systematic surveillance of harms**	Collection of *adverse outcomes* that does not use a systematic approach (i.e., researchers do not ask about specific *adverse outcomes* or apply standardised testing). This approach usually involves asking participants to report the presence of *adverse outcomes* they have noticed (this can be through active or passive surveillance).
**Passive surveillance of harms**	Participants are not specifically asked about or tested for the occurrence of *adverse outcome*. Rather, *adverse outcomes* are identified based on participant or exercise professional reports made on their own initiative. This is also called spontaneous reporting.
**Persistent adverse outcome**	A *persistent adverse outcome* is an *adverse outcome* that extends continuously, without resolution.<br>The *adverse outcome* is reported only once unless the grade changes. If the grade becomes more or less severe the *adverse outcome* is reported again, as a new *adverse outcome* with the new grade.
**Recall period**	The period between each occasion of *adverse outcome* reporting (by participant or clinician).<br>E.g., If the exercise professional asks the participant to report any *adverse outcomes* that have occurred since the previous session that was 7 days ago, then the *recall period* would be 7 days.
**Recurrent adverse outcome**	A *recurrent adverse outcome* is an *adverse outcome* that occurs and resolves.<br>An *adverse outcome* that resolves and then recurs should be reported at each recurrence.
**Serious adverse event**	The term *Serious adverse event* has not been used in in ExHaRM. We acknowledge the term here as it is often a reporting requirement of ethics or institutional boards.<br><br>*Serious adverse events* are *adverse events* that meet a specific definition, separate to severity. The term is primarily used in defining regulatory reporting obligations for clinical trials. In ExHaRM *adverse events* are captured by the umbrella term *adverse outcome. Adverse outcomes* are divided by grade into mild-moderate (grade 1-2) and severe (≥ grade 3), rather than being defined as serious and non-serious.<br><br>*Serious adverse events*[14] are any exercise-related *adverse events* (i.e., "undesirable medical occurrences") occurring at any dose of an intervention that results in ANY of the following outcomes:<br>1) Death.<br>2) A life-threatening experience.<br>3) Inpatient hospitalization or prolongation of existing hospitalization (for >24 hours).<br>4) A persistent or significant incapacity or substantial disruption of the ability to conduct normal life functions<br>5) A congenital anomaly/birth defect.<br>6) Important Medical Events that may not result in death, be life threatening, or require hospitalization may be considered a serious adverse experience when, based upon medical judgment, they may jeopardize the patient or subject and may require medical or surgical intervention to prevent one of the outcomes listed in this definition<br><br>Reporting requirements are usually determined by ethics or institutional boards and in most cases there are specific reporting requirements for *serious adverse events*. Given clinical and legal implications of these events, it is

For peer review only

	recommended that all exercise interventions and programs have a process requiring exercise professionals to report these *serious adverse events* to senior staff. Further, it is recommended that any *adverse outcomes* that meet the above definition should be reported to supervisors/ senior staff for review, regardless of whether the exercise professional judged the event to be exercise-related. It is recommended that *serious adverse events* (or *adverse outcomes* where *causality* could not be confidently assessed, but that otherwise met any serious adverse event criteria) are reported to the harms panel within 48 hours for immediate review.
**Severity / Grade**	*Adverse outcomes* are given a severity rating based on a grading scale of 1-5 (as per CTC-AE).<br><br>Severity may also be grouped into categories of mild-moderate (grade 1-2 *adverse outcomes*) or severe (grade 3-5 *adverse outcomes*).<br>Note: A severe *adverse outcome*, is NOT the same as *serious adverse event* which is defined separately (see "*serious adverse events*")
**Grade 1**	Mild; asymptomatic or mild symptoms; clinical or diagnostic observations only; medical treatment not indicated.<br><br>*ExHaRM application: "no/minimal disruption to activities of daily living"*
**Grade 2**	Moderate; minimal, local or noninvasive medical treatment indicated; limiting age-appropriate instrumental activities of daily living.<br>[Instrumental activities of daily living refer to activities such as preparing meals, shopping for groceries, using the telephone, managing money, etc.]<br><br>*ExHaRM application: "some disruption to instrumental activities of daily living"*
**Grade 3**	Severe or medically significant but not immediately life-threatening; hospitalization or prolongation of hospitalization indicated; disabling; substantially limiting self-care activities of daily living.<br>[Self-care activities of daily living refer to activities including bathing, dressing and undressing, feeding self, using the toilet, taking medications.]<br><br>*ExHaRM application: "complete disruption to self-care activities of daily living"*
**Grade 4**	Life-threatening consequences; urgent medical treatment indicated.
**Grade 5**	Death related to *adverse outcome*.

For peer review only - http://bmjopen.bmj.com/site/about/guidelines.xhtml

**Systematic surveillance of harms**	Asking every participant (at defined time points) about specific *adverse outcomes or* performing specific laboratory or other diagnostic tests. Systematic surveillance is, by definition, one approach to active surveillance of harms.<br><br>Systematic methods, such as querying participants using a comprehensive checklist or standardized laboratory tests, are more likely than non-systematic methods to identify all occurrences of an *adverse outcome*.
**Type of adverse outcome**	Relevant categories of *Type of adverse outcome* may depend on the type of intervention and any *harms of interest* collected.<br><br>*Example list and reporting codes*<br>**Ex:** normal response to exercise (e.g., DOMS),<br>**AbEx:** abnormal response to exercise,<br>**SE:** expected disease- or treatment-related side effect,<br>**SE+:** exacerbation of disease- or treatment-related side effect,<br>**In:** injury,<br>**O:** other (describe)

For peer review only

Considerations and future directions

Exercise is frequently reported as being safe for those diagnosed with cancer. Yet quality data supporting this statement is lacking. ExHaRM presents an exercise harms reporting protocol ready for widespread use, providing practical, exercise-specific guidelines for the collection and reporting of harms in exercise oncology research. In line with best practice,[23] ExHaRM facilitates reporting of any, and all, adverse outcomes, of any severity and causality. In doing so, ExHaRM supports collection of exercise-related adverse outcomes required for quantifying harms of exercise, while concurrently (i) collects information necessary for targeted, individualised exercise prescription; (ii) reduces the influence of potential participant or exercise professional bias on harms reporting; (iii) allows for exploring the rate of exercise-related adverse outcomes to all-cause adverse outcomes, which may help identify attribution bias;[23] and (iv) provides the necessary data for inclusion in future meta-analyses to determine the strength of harms evidence in exercise oncology and to evaluate whether specific patient (e.g., age, cancer type, stage of disease at diagnosis) or exercise characteristics (e.g., frequency, intensity, mode, mode of delivery) influence harms.

We recognise that just as is the case for assessing potential exercise benefit, the goal for future ExHaRM iterations will be to reduce data collection burden (to participants, researchers, funding bodies etc) while maintaining comprehensive collection of meaningful information about exercise harms. Integrating a checklist of expected and clinically-important exercise-related harms (e.g., similar to the Common Terminology Criteria for Adverse Events[11] and the associated patient-reported version (PRO-CTCAE)[24]) is a proposed development for future iterations of ExHaRM. However, while this may prove useful in streamlining harms data collection, it would not replace the need to collect adverse outcomes through more time-consuming non-systematic approaches as promoted in the current ExHaRM version.

The identification of those adverse outcomes that are exercise-related is essential to the evaluation of the harms of exercise. The validity of the causality assessment process described in ExHaRM has not been formally evaluated and relies on experienced exercise and medical clinicians being involved, at a minimum, in the reviewing of causality attribution. Development of an algorithm similar to the causality probability scale developed by Naranjo et al[25] would be one approach to standardising causality attribution in exercise oncology. However, in the interim, ExHaRM provides clear guidance to exercise

For peer review only - http://bmjopen.bmj.com/site/about/guidelines.xhtml

professionals in judging the exercise-relatedness of adverse outcomes. We have found the involvement of a harms panel in ExHaRM extremely useful in reducing the potential for over- and under-reporting exercise-related adverse outcomes and ensuring consistency in the classification of adverse outcomes.

ExHaRM does not include a threshold at which exercise would be defined safe or unsafe. Decisions regarding the role of exercise in a specific context should be based on weighing evidence of the expected benefits against the potential harms. As is true with medical treatments, in some situations the benefits are sufficient that a higher level of concurrent harm is deemed acceptable. For example, life-saving chemotherapy is recommended despite the expectation of extensive undesirable consequences. Within exercise oncology, some patient groups (e.g., those with advanced bone lesions or metastases) may be at higher risk of harm from exercise; however, if the benefits were of sufficient value to the population (e.g., maintained ability to independently complete activities of daily living) then exercise may be incorporated among care despite the harm profile.

With iterations of ExHaRM there was increasing recognition of the need to identify and consistently apply appropriate terms. Much discussion and deliberation has occurred over the use of all harms-related terms, but in particular the use of harms versus safety (which presupposes a positive outcome), and adverse outcomes versus adverse events. These discussions acknowledged that there are unintended negative consequences (i.e., harms) of changing lifestyle behaviours, that do not meet the definition of an adverse event as used in pharmacological harms reporting.[12] Adverse outcome (as used in the current iteration of ExHaRM) is a widely encompassing term which addresses the limitations of the term adverse event. There was also deliberation of whether adverse outcomes classified with the causality category of 'possible' should be defined, along with those specified as 'certain' and 'likely', as exercise-related adverse outcomes. This issue of causality categorisation also remains an area of debate in the harms reporting of pharmacological clinical trials.[26] Our current approach to include 'possible' is more inclusive, which was considered appropriate given the infancy of this specific area of harms reporting, but is a topic for future discussion and debate.

For peer review only - http://bmjopen.bmj.com/site/about/guidelines.xhtml

ExHaRM was developed for use in research. However, with minimal modifications this protocol could also be implemented in hospital-, university- and community-based exercise settings. Monitoring adverse outcomes (and near misses, in which no adverse outcome occurred but could have) within a clinic would facilitate risk mitigation through modification of processes (e.g., modification of prescription, type of equipment used, inclusion of contraindications or conditions that should be pre-screened), with the goal of reducing harms without compromising benefit. The collection of harms data in real-world settings also allows for the identification of rare adverse outcomes not previously identified during research, as well as those that may only occur outside of controlled research settings. Further, this harms reporting framework may also prove useful in other lifestyle intervention trials beyond exercise.

Summary

There is a clear imperative to improve harms reporting in exercise oncology, and this imperative is heightened with the inclusion of understudied, rare cancers, and potentially higher-risk cohorts and interventions (e.g., participants with poorer prognoses and/or extensive comorbidities; higher intensity and/or less supervised exercise interventions) in exercise trials. In the short-term, as per CONSORT guidelines,[10, 27] all exercise trials should have a harms reporting protocol. In the longer term, consensus regarding a standardised protocol and consistent use of harms-related terms, such as adverse outcomes, will facilitate rapid progress in this specific area of exercise oncology research.

For peer review only

For peer review only - http://bmjopen.bmj.com/site/about/guidelines.xhtml

Acknowledgements

The authors would like to acknowledge the contribution of participants, exercise professionals and chief investigators who were part of the SAFE, BRACE, ECHO and ECHO-R trials that were fundamental in the development of ExHaRM.

Authors' Contributions:

RRS: Conceptualization, Methodology, Investigation, Data curation, Writing – Original Draft. CXS: Investigation, Data curation, Writing – Review & Editing. TMJ: Data curation, Writing – Review & Editing. NMM: Visualization, Writing – Review & Editing. RMD: Investigation, Writing – Review & Editing. SCH: Conceptualization, Supervision, Data curation, Writing – Review & Editing, Funding acquisition.

Funding statement:

The suite of ihop exercise oncology trials that have contributed to the work presented have received funding from the following bodies: Icon Cancer Foundation (BRACE trial), Medical Research Future Fund (ECHO-R trial), and Cancer Australia, Cancer Council Australia, World Cancer Research Fund, Cancer Council Queensland and Griffith University (ECHO trial). CXS is supported by a Cancer Institute New South Wales Early Career Fellowships (2021/ECF1310).

Competing Interests statement.

The authors declare that they have no competing interests.

For peer review only - http://bmjopen.bmj.com/site/about/guidelines.xhtml

1. Hayes, S.C., et al., *Australian Association for Exercise and Sport Science position stand: optimising cancer outcomes through exercise.* J Sci Med Sport, 2009. **12**(4): p. 428-34.
2. Schmitz, K.H., et al., *American College of Sports Medicine roundtable on exercise guidelines for cancer survivors.* Med Sci Sports Exerc, 2010. **42**(7): p. 1409-26.
3. Campbell, K.L., et al., *Exercise Guidelines for Cancer Survivors: Consensus Statement from International Multidisciplinary Roundtable.* Med Sci Sports Exerc, 2019. **51**(11): p. 2375-2390.
4. Hayes, S.C., et al., *The Exercise and Sports Science Australia position statement: Exercise medicine in cancer management.* J Sci Med Sport, 2019. **22**(11): p. 1175-1199.
5. Reginster, J.Y., et al., *Importance of Safety in the Management of Osteoarthritis and the Need for Updated Meta-Analyses and Recommendations for Reporting of Harms.* Drugs Aging, 2019. **36**(Suppl 1): p. 3-6.
6. Adams, S.C., et al., *Comparing the reporting and conduct quality of exercise and pharmacological randomised controlled trials: a systematic review.* BMJ Open, 2021. **11**(8): p. e048218.
7. Niemeijer, A., et al., *Adverse events of exercise therapy in randomised controlled trials: a systematic review and meta-analysis.* Br J Sports Med, 2020. **54**(18): p. 1073-1080.
8. Singh, B., et al., *Exercise and colorectal cancer: a systematic review and meta-analysis of exercise safety, feasibility and effectiveness.* Int J Behav Nutr Phys Act, 2020. **17**(1): p. 122.
9. Singh, B., et al., *A systematic review and meta-analysis of the safety, feasibility and effect of exercise in women with stage II+ breast cancer.* Archives of Physical Medicine and Rehabilitation, 2018. **https://doi.org/10.1016/j.apmr.2018.03.026**.
10. Ioannidis, J.P., et al., *Better reporting of harms in randomized trials: an extension of the CONSORT statement.* Ann Intern Med, 2004. **141**(10): p. 781-8.
11. National Cancer Institute (U.S.), *Common terminology criteria for adverse events (CTCAE) - version 5.0*. NIH publication. 2017, Bethesda, Md.: U.S. Dept. of Health and Human Services, National Institutes of Health, National Cancer Institute.
12. Papaioannou, D., et al., *Adverse event recording failed to reflect potential harms: a review of trial protocols of behavioral, lifestyle and psychological therapy interventions.* J Clin Epidemiol, 2021. **136**: p. 64-76.
13. INTERNATIONAL COUNCIL FOR HARMONISATION OF TECHNICAL REQUIREMENTS FOR PHARMACEUTICALS FOR HUMAN USE (ICH), *Indexed ICH GCP guidelines with integrated addendum E6(R2), Step 4*. First edition. ed, ed. D.R. Hutchinson. 2016, Chobham, Surrey: International council for harmonisation. 64 pages.
14. National Institutes of Health (NIH), *NCI Guidelines For Investigators: ADVERSE EVENT REPORTING REQUIREMENTS*, U.S. Dept. of Health & Human Services, Editor. 2013.
15. National Health and Medical Research Council, *Guidance: Safety monitoring and reporting in clinical trials involving therapeutic goods*. 2016, National Health and Medical Research Council.: Canberra.
16. Gildea, G., Sandler CX., Elisadis, P., Pickersgill, D., Shevill, M., Bigg, V., Morrison, B., Bashford, J., Jonker, F., Hayes, S., *Evaluating exercise for patients with brain cancer – The BRACE study.* Asia-Pacific Journal of Clinical Oncology, 2021. **17**(S9).

For peer review only - http://bmjopen.bmj.com/site/about/guidelines.xhtml

17. RR, S., et al., *A Randomised, Comparative, Effectiveness Trial Evaluating Low- versus High-Level Supervision of an Exercise Intervention for Women with Breast Cancer: The SAFE Trial.* Cancers, 2022. **14**(6).
18. Hayes S, F.M., Obermair A, Mileshkin L, Janda M, Gordon L, Barnes E, Beesley V, Eakin E, Sommeijer D, Martyn J, Stockler M, Gebski VAL, Naumann F, Schmitz K, Webb P, *Exercise during chemotherapy for ovarian cancer (ECHO): study design features and outcomes of a cancer Australia and cancer council australia funded randomised, controlled trial | Cochrane Library.* International journal of gynecological cancer, 2014. **24**: p. 200-201.
19. Sandler, C., et al., *Exercise during Chemotherapy for Recurrent Ovarian Cancer (ECHO-R): A Phase II trial evaluating feasibility, safety and efficacy of an individually-tailored exercise intervention during chemotherapy for recurrent ovarian cancer* Asia-Pacific Journal of Clinical Oncology, 2022. **Accepted**.
20. International Council for Harmonisation of Technical Requirements for Pharmaceuticals for Human Use, *MedDRA® Medical Dictionary for Regulatory Activities terminology. Retrieved from https://www.meddra.org/*. 2020.
21. Kelly, W. and S. Halabi, *Oncology Clinical Trials: Successful Design, Conduct, and Analysis*. 2nd ed. 2018.
22. World Health Organization (WHO)-Uppsala Monitoring Centre *The use of the WHO-UMC system for standardized case causality assessment*.
23. Enrico, D., et al., *Analysis of adverse events attribution and reporting in cancer clinical trials: A systematic review.* Crit Rev Oncol Hematol, 2021. **160**: p. 103296.
24. Kluetz, P.G., et al., *Patient-Reported Outcomes in Cancer Clinical Trials: Measuring Symptomatic Adverse Events With the National Cancer Institute's Patient-Reported Outcomes Version of the Common Terminology Criteria for Adverse Events (PRO-CTCAE).* Am Soc Clin Oncol Educ Book, 2016. **35**: p. 67-73.
25. Naranjo, C.A., et al., *A method for estimating the probability of adverse drug reactions.* Clin Pharmacol Ther, 1981. **30**(2): p. 239-45.
26. Pande, S., *Causality or Relatedness Assessment in Adverse Drug Reaction and Its Relevance in Dermatology.* Indian J Dermatol, 2018. **63**(1): p. 18-21.
27. Schulz, K.F., et al., *CONSORT 2010 statement: updated guidelines for reporting parallel group randomised trials.* BMJ, 2010. **340**: p. c332.

For peer review only

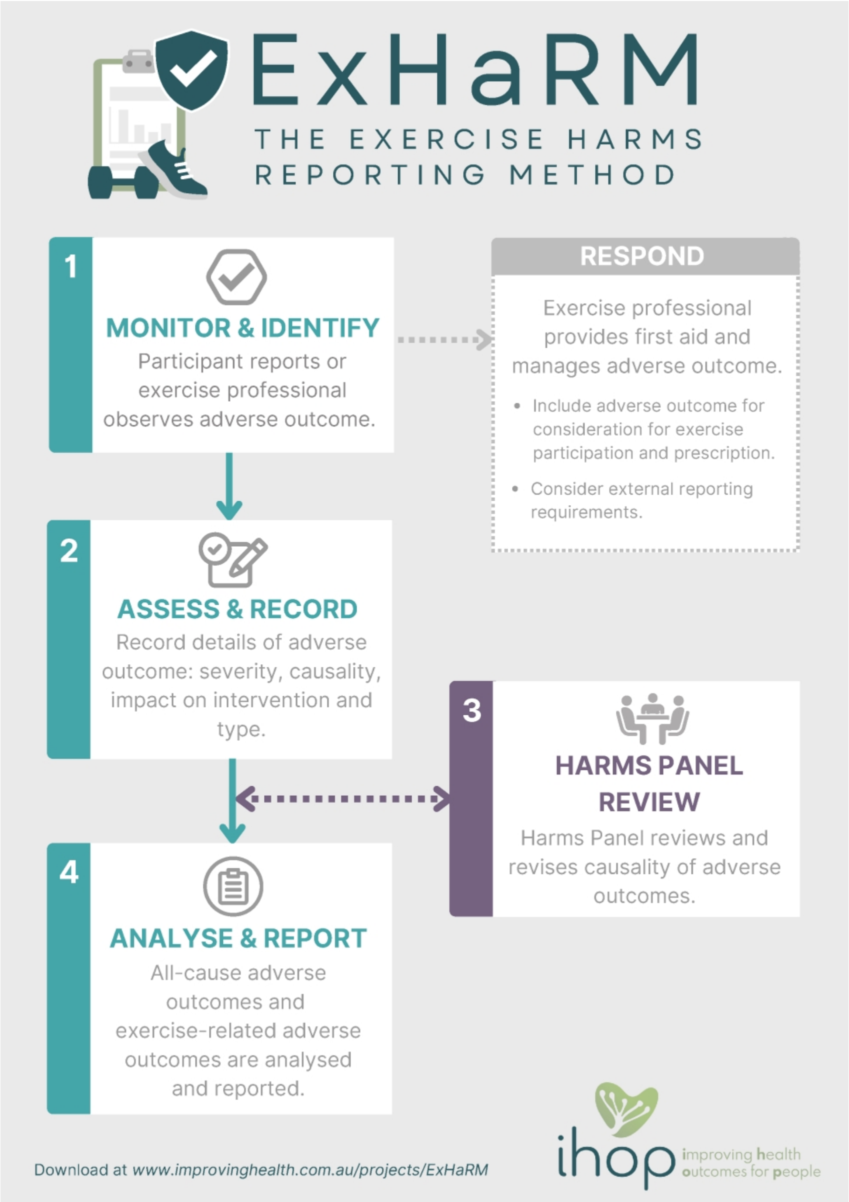

Figure 1: An overview of the Exercise Harms Reporting Method (ExHaRM)
Notes: See Table 1 for definitions and further explanation of terms used in this flow chart.

210x297mm (300 x 300 DPI)

Practical suggestions for harms reporting in exercise oncology: the Exercise Harms Reporting Method (ExHaRM)

Journal:	*BMJ Open*
Manuscript ID	bmjopen-2022-067998.R2
Article Type:	Communication
Date Submitted by the Author:	10-Nov-2022

Complete List of Authors:	Spence, Rosalind; Griffith University, Menzies Institute of Health Queensland; Griffith University, Improving Health Outcomes for People (ihop) research group, School of Health Sciences and Social Work<br>Sandler, Carolina; Griffith University, Improving Health Outcomes for People (ihop) research group, School of Health Sciences and Social Work; Western Sydney University, Sport and Exercise Science, School of Health Science<br>Jones, Tamara; Griffith University, Improving Health Outcomes for People (ihop) research group, School of Health Sciences and Social Work; University of Melbourne, Melbourne School of Psychological Sciences, Faculty of Medicine, Dentistry, and Health Sciences<br>McDonald, Nicole; Griffith University, Menzies Health Institute Queensland; Griffith University, Improving Health Outcomes for People (ihop) research group, School of Health Sciences and Social Work<br>Dunn, Riley; Griffith University, Menzies Health Institute Queensland; Griffith University, Improving Health Outcomes for People (ihop) research group, School of Health Sciences and Social Work<br>Hayes, Sandra; Griffith University, Menzies Health Institute Queensland; Griffith University, Improving Health Outcomes for People (ihop) research group, School of Health Sciences and Social Work

**Primary Subject Heading**:	Sports and exercise medicine
Secondary Subject Heading:	Oncology
Keywords:	SPORTS MEDICINE, PUBLIC HEALTH, ONCOLOGY

SCHOLARONE™ Manuscripts

I, the Submitting Author has the right to grant and does grant on behalf of all authors of the Work (as defined in the below author licence), an exclusive licence and/or a non-exclusive licence for contributions from authors who are: i) UK Crown employees; ii) where BMJ has agreed a CC-BY licence shall apply, and/or iii) in accordance with the terms applicable for US Federal Government officers or employees acting as part of their official duties; on a worldwide, perpetual, irrevocable, royalty-free basis to BMJ Publishing Group Ltd ("BMJ") its licensees and where the relevant Journal is co-owned by BMJ to the co-owners of the Journal, to publish the Work in BMJ Open and any other BMJ products and to exploit all rights, as set out in our licence.

The Submitting Author accepts and understands that any supply made under these terms is made by BMJ to the Submitting Author unless you are acting as an employee on behalf of your employer or a postgraduate student of an affiliated institution which is paying any applicable article publishing charge ("APC") for Open Access articles. Where the Submitting Author wishes to make the Work available on an Open Access basis (and intends to pay the relevant APC), the terms of reuse of such Open Access shall be governed by a Creative Commons licence – details of these licences and which Creative Commons licence will apply to this Work are set out in our licence referred to above.

Other than as permitted in any relevant BMJ Author's Self Archiving Policies, I confirm this Work has not been accepted for publication elsewhere, is not being considered for publication elsewhere and does not duplicate material already published. I confirm all authors consent to publication of this Work and authorise the granting of this licence.

For peer review only

Practical suggestions for harms reporting in exercise oncology: the Exercise Harms Reporting Method (ExHaRM)

Rosalind R Spence[1, 2], Carolina X Sandler[2, 3], Tamara L Jones[2, 4], Nicole M McDonald[1, 2], Riley M Dunn[1, 2], Sandra C Hayes[1, 2]

[1] Menzies Health Institute Queensland, Griffith University, Brisbane, Australia

[2] Improving Health Outcomes for People (ihop) research group, School of Health Sciences and Social Work, Griffith University, Brisbane, Australia

[3] Sport and Exercise Science, School of Health Science, Western Sydney University, Sydney, NSW, Australia.

[4] Melbourne School of Psychological Sciences, Faculty of Medicine, Dentistry, and Health Sciences, University of Melbourne

Word Count: 3150
Online supplemental material: 1
Figures: 1

Corresponding author: Rosalind R Spence
Email: r.spence@griffith.edu.au
Postal: Menzies Health Institute Queensland, Griffith 43 University, Brisbane, Australia.
Postal address: Griffith University Nathan Campus, 170 Kessels Road, 44 NATHAN, QLD 4111

Keywords: cancer; adverse events; clinical trial reporting; safety; harms

For peer review only

Abstract

The volume of high-quality evidence supporting exercise as beneficial to cancer survivors has grown exponentially; however, the potential harms of exercise remain understudied. Consequently, the trade-off between desirable and undesirable outcomes of engaging in exercise remains unclear to clinicians and people with cancer. Practical guidance on collecting and reporting harms in exercise oncology is lacking. We present a harms reporting protocol developed and refined through exercise oncology trials since 2015.

Development of the Exercise Harms Reporting Method (*ExHaRM)* was informed by national and international guidelines for harms reporting in clinical trials involving therapeutic goods or medical devices, with adaptations to enhance applicability to exercise. The protocol has been adjusted via an iterative process of implementation and adjustment through use in multiple exercise oncology trials involving varied cancer diagnoses (types: breast, brain, gynaecological; stages at diagnosis I-IV; primary/recurrent), and heterogeneous exercise intervention characteristics (face-to-face/telehealth delivery; supervised/unsupervised exercise). It has also involved the development of terms (such as, adverse outcomes, which capture all undesirable physical, psychological, social and economic outcomes) that facilitate the harms assessment process in exercise.

ExHaRM involves: Step 1: Monitor occurrence of adverse outcomes through systematic and non-systematic surveillance; Step 2: Assess and record adverse outcomes, including severity, causality, impact on intervention and type; Step 3: Review of causality by harms panel (and revise as necessary); and Step 4: Analyse and report frequencies, rates and clinically meaningful details of all-cause and exercise-related adverse outcomes.

ExHaRM provides guidance to improve the quality of harms assessment and reporting immediately, while concurrently providing a framework for future refinement. Future directions include, but are not limited to, standardising exercise-specific nomenclature and methods of assessing causality.

For peer review only

Introduction

Since publication of the first exercise oncology prescription guidelines over a decade ago, [1, 2] the volume and quality of evidence supporting the benefits of exercise following a cancer diagnosis has increased exponentially.[3, 4] The current model of evidence-based medicine is founded on the principle that expected benefits, as well as potential harms, patient values and preferences, drive clinician recommendations and patient decisions.[5] Unfortunately, the harms of exercise remain understudied and underreported in exercise trials (missing or incompletely reported in 51-75% of studies),[6, 7] and specifically, within exercise oncology (not reported in 34-47% of studies).[8, 9] Harms are "the totality of possible adverse consequences of an intervention or therapy; they are the direct opposite of benefits, against which they must be compared."[10] The absence of data regarding the harms of exercise significantly compromises the ability of clinicians, patients and exercise professionals to evaluate the trade-off between desirable and undesirable consequences.[5] There is insufficient evidence for (i) clinicians to judge the safety profile of exercise as a potential adjuvant therapy, (ii) patients to make informed decisions about potential consequences of exercise participation, and (iii) exercise professionals to determine the appropriateness of exercise (i.e., contraindications, or dosage and prescription considerations).

Despite a growing recognition of the importance of accurate and comprehensive reporting of the harms of exercise,[7] practical guidance on how to manage the collection and reporting process in exercise oncology interventions is missing.[8, 9] Well-accepted standards and guidelines for harms reporting in pharmacological cancer clinical trials exist.[11] However, the direct application of these frameworks to exercise trials, without modification, is inappropriate.[12] Pharmacological harms are assessed via adverse events (i.e., "any undesirable medical occurrence" [13]). However, the collection of only adverse events fails to capture the breadth of potential negative consequences that may occur due to behavioural interventions, including exercise.[12] Harms reporting in exercise interventions is further complicated by the need to distinguish harms associated with medical treatment from those associated with exercise. Below we share our approach to exercise harms reporting, with the aim of encouraging more comprehensive reporting of harms data and providing a platform and framework from which future studies can build.

Exercise Harms Reporting Method

For peer review only - http://bmjopen.bmj.com/site/about/guidelines.xhtml

The **Ex**ercise **Ha**rms **R**eporting **M**ethod (ExHaRM) is a protocol for the monitoring and reporting of exercise-related harms. It was developed based on guidelines for harms reporting in clinical trials involving therapeutic goods or medical devices (specifically, Good Clinical Practice guidelines;[13] National Cancer Institute Common terminology criteria for AEs [CTC-AE][11]), as well as international,[14] Australian and local institutional reporting requirements,[15] and the expert opinions of trial investigators (including exercise oncology researchers, accredited exercise physiologists, cancer nurses, breast surgeons, gynaecological oncologists and medical oncologists). Our team first implemented an intentional harm assessment and reporting method in 2015, with the commencement of exercise oncology trials targeting cancer survivors with multiple comorbidities[16] or undergoing intense treatment,[17] who were potentially higher-risk exercisers than those included in previous trials. Since that time, the protocol has been refined through an iterative process of implementation and adjustment. Iterations of ExHaRM have been used in four exercise oncology trials,[16-19] involving samples with breast, brain and gynaecological cancer, different stages of disease at diagnosis (stage I-IV), primary and recurrent diagnoses, and the presence of multiple comorbidities. Further, ExHaRM has been used in studies that involve mixed-mode exercise (aerobic and resistance training) delivered during and following treatment, via various modes of delivery (face-to-face supervision ranging from 2 sessions/week to 1 session/month, in addition to trials delivered via tele-health), and a range of settings (e.g., exercise completed at participants' homes, community fitness centres, and university exercise clinics).

ExHaRM defines the harms of exercise as all undesirable physical, psychological, economic or social consequences (covering incidences, experiences, occurrences) that are related to an individual's participation in exercise. Given assessment of **adverse events** does not fully capture harms of exercise, we therefore propose the use of the term **adverse outcomes** to assess undesirable physical, psychological, social and economic outcomes, experiences or events, irrespective of whether they have a causal relationship with exercise. ExHaRM involves four core steps (summarised in Figure 1 and explained below) to capture, classify, analyse and report all-cause and exercise-related adverse outcomes. A downloadable and interactive version of ExHaRM is available at www.improvinghealth.com.au/projects/exharm). Key terms, including their definitions and related information (e.g., prompts or questions to ensure accurate data collection, and coding

For peer review only - http://bmjopen.bmj.com/site/about/guidelines.xhtml

recommendations to aid efficient recording) are denoted by italics throughout the method and can be in online supplemental material Box 1.

Details of the reporting protocol

The **Ex**ercise **Ha**rms **R**eporting **M**ethod (ExHaRM)

The purpose of this protocol is to guide the collection and reporting of informative data on the *harms of exercise*.

Note: Italicised terms are defined in box 1 (online supplemental material).

1) Monitor & Identify

Systematically monitor and identify *adverse outcomes*:

- Actively prompt participants to report *adverse outcomes*. Use standardised language and standardised *recall periods*. For example, the exercise professional asks the participant at every contact: "Since our last session have you experienced any harmful or undesirable outcomes, whether or not you think they were caused by exercise? For example, have you had an injury or worsening of an old injury, or any unusual or worsening treatment-related symptom?" (i.e., *active surveillance*)
- Train exercise professionals to actively observe for *adverse outcomes* that may occur during supervised exercise, and to probe during discussions if a participant reports something that might be an *adverse outcome* (i.e., *passive surveillance* / spontaneous reporting of *adverse outcomes*).
- Consider generating a study-specific list of *adverse outcomes of interest* that are systematically recorded via a checklist or standardised test at fixed time points (*systematic surveillance*), in addition to the broad question recommended above (*non-systematic surveillance*).

Respond

Whilst not part of the harms-reporting process, the exercise professional has a duty of care to the participant to identify, consider and manage all *adverse outcomes* and depending on the nature of the *adverse outcome* (with particular attention required for *adverse events*) may have external reporting requirements:

For peer review only

- Provide first aid and management of the *adverse outcome*: Respond to immediate needs of the participant through administration of first aid, cessation or modification of exercise, referral to other health professional, and/or other responses as required.
- Complete tasks as per exercise professional duties and scope of practice: Consider whether *adverse outcome* requires an absence from exercise (i.e., is it a contraindication?) and/or modification of exercise parameters (e.g., volume, intensity, or type).
- Report as per requirements of employer, institution, funding and / or ethics committees.

  Note: The definitions (e.g., of *harms*, *adverse outcomes*, and *severity*) in this protocol are those determined to be most appropriate to the evaluation of exercise-related harms. Regulatory committees (e.g., a human research ethics committee) typically require the reporting of "*serious adverse events*" only, which will be captured within the monitoring and reporting of *adverse outcomes* as outlined in this protocol.

2) Assess & Record

Assess and record the *adverse outcome* and associated classifications of *severity, causality, impact of adverse outcome on the intervention,* and *type of adverse outcome,* including sufficient detail to justify the assigned category. Also record detail of the context and sequalae of the *adverse outcome*; for example, where and when the *adverse outcome* occurred, what clinical action was taken, the duration of the *adverse outcome* and any subsequent treatment.

Severity (Grade 1-5): based primarily on the resultant medical treatment and the impact of the *adverse outcome* on activities of daily living.

Causality: based on the timing of the *adverse outcome* in relation to exercise and plausibility of relationship with exercise or other factors (see useful questions to consider under "*Causality*"). Include both the participant's opinion and exercise professional's judgement of *causality*.

Impact on intervention: based on changes to a participant's involvement in the exercise intervention caused by the *adverse outcome*. This may involve

For peer review only

modifications (e.g., modified exercise prescription, missed exercise sessions), or absences from the intervention (either temporary or permanent).

Type of adverse outcome: the purpose of categorising *adverse outcome* by type is to create groupings for simplified summary based on predetermined categories (e.g., abnormal response to exercise, injury, exacerbation of treatment-related side effect).

Note: Care should be taken to differentiate between a *persistent* and *recurrent adverse outcome*, as *persistent adverse outcomes* need only be reported once, whereas *recurrent adverse outcomes* should be reported at each recurrence.

3) Review by *Harms Panel* and potential revision of causality

The attribution of *causality* (i.e., determining which *adverse outcomes* are causally related to exercise) is essential to the evaluation of exercise-related harms. *Causality* attribution requires knowledge of the mechanisms by which an intervention may cause harm, as well as the expected responses to an intervention (i.e., scope of exercise professional). Concurrently, an understanding of what is common and expected in the population (both disease- and treatment-related) is also required and is likely most appropriately provided by the medical team. Therefore, while it is appropriate for exercise professionals to make the initial judgement of causality, ideally *adverse outcomes* should be reviewed by a panel with additional expertise, and preferably at least one individual who is not directly involved in the study.

All *adverse outcomes* are reported to the study *harms panel* on a pre-determined schedule. This may be frequently (e.g., monthly) or at a single point prior to data analysis.

The *harms panel* review all *adverse outcomes* with special attention to:

- unusual or unanticipated *adverse outcomes* (as judged by panel);
- discordant opinions of causality recorded between participant and exercise professional;
- *adverse outcomes* ≥ grade 3.
- *adverse outcomes* that the exercise professional has highlighted for review

For peer review only - http://bmjopen.bmj.com/site/about/guidelines.xhtml

Discrepancies in *causality* attribution between exercise professional and any member of the harms panel should be discussed, and the consensus reached by the panel be recorded as the final *causality* decision.

The *harms panel* may also determine that a reported *adverse outcome* does not meet the definition of an *adverse outcome* (e.g., the recorded event might not be judged by the panel as being undesirable). In this situation the *adverse outcome* would be removed from the dataset and would not be reported.

4) Analyse & Report

4.1 Data preparation and analysis

- Any *adverse outcome* with a *causality* of certain, likely, or possible is categorised as an *exercise-related adverse outcome*. The term *"all-cause adverse outcomes"* is then used to refer to *adverse outcome* of any *causality* (i.e., all *adverse outcomes*: those deemed exercise related, as well as those not attributed to exercise).
- Re-code *adverse outcomes* with standardised language: each *adverse outcome* identified in the study should be described using standardised language (e.g., *Medical dictionary for Regulatory Activities* [MedDRA®][20] preferred terms) and summarised (e.g., within system organ class or appropriate groupings). For example: a report of "stiff and sore shoulders" as an *adverse outcome* in the clinical notes could be coded as "arthralgia" using *MedDRA terminology* and listed under the category of "Musculoskeletal and Connective Tissue Disorders", along with other conditions, such as arthritis.
- Group all-cause *adverse outcomes* and *exercise-related adverse outcomes* by each category of *Type of Adverse outcome* and *Impact on intervention*.

4.2 Reporting

All-cause and *Exercise-related adverse outcomes:*

- Report the number of *all-cause adverse outcomes* and *exercise-related adverse outcomes*, including the number (and proportion) of *all-cause adverse outcomes* and *exercise-related adverse outcomes* categorised by *severity* as mild-moderate (grades 1 and 2) and severe (grades 3-5).
- For these harms outcomes (i.e., number of all-cause *adverse outcomes* and *exercise-related adverse outcomes* by severity categories) it is helpful to also report the

For peer review only - http://bmjopen.bmj.com/site/about/guidelines.xhtml

number of participants (and proportion of all participants) reporting an *adverse outcome* at least once.

- The number of *adverse outcomes* (and percentage) for each MedDRA code (e.g., arthralgia) and system organ class (e.g., musculoskeletal and connective tissue disorders), or other standardised language groupings, should be reported for all-cause *adverse outcomes* and for *exercise-related adverse outcomes*.

Exercise-related adverse outcomes only*:*

- The number (and proportion) of *exercise-related adverse outcomes* in each category of '*impact on intervention*' and '*type of adverse outcome*' should be reported.
- Highlight any unique or key *exercise-related adverse outcomes* relevant to the context of the study, or of clinical relevance (e.g., lymphoedema, dislodged peripherally-inserted central catheter, bone fractures).
- Consider reporting *exercise-related adverse outcomes* as a rate per person per week of intervention, in conjunction with the average weekly minutes of exercise that were completed. If rate is not reported, then ensure sufficient intervention and trial details are included to allow subsequent studies to compare results.

- *Adverse outcomes* that occur during exercise testing (i.e., objective data collection) should be reported separately to those that occur during the intervention.

Considerations and future directions

Exercise is frequently reported as being safe for those diagnosed with cancer. Yet quality data supporting this statement is lacking. Our understanding of the need to collect harms data, and subsequently, optimal methods for assessing and reporting harms has evolved. For a decade before the first iteration of ExHaRM, our trials involved intentional collection of disease- and treatment-related side effects, as well as undesirable responses to exercise, for the purpose of exercise prescription modification. With the advent of trials including participants for whom exercise was potentially more likely to cause undesirable outcomes, and exploring less controlled exercise settings, recording and reporting safety-related data became a higher priority. Through these trials, ExHaRM progressed to include systematic recording and reporting of adverse events, the assessment of additional relevant information (e.g., causality,

For peer review only - http://bmjopen.bmj.com/site/about/guidelines.xhtml

severity, impact) and then exercise-specific nomenclature and definitions (e.g., adverse outcomes, as defined in the current version).

ExHaRM now presents an exercise harms reporting protocol ready for widespread use, providing practical, exercise-specific guidelines for the collection and reporting of harms in exercise oncology research. In line with best practice,[21] ExHaRM facilitates reporting of any, and all, adverse outcomes, of any severity and causality. In doing so, ExHaRM supports collection of exercise-related adverse outcomes required for quantifying harms of exercise, while concurrently (i) collects information necessary for targeted, individualised exercise prescription; (ii) reduces the influence of potential participant or exercise professional bias on harms reporting; (iii) allows for exploring the rate of exercise-related adverse outcomes to all-cause adverse outcomes, which may help identify attribution bias;[21] and (iv) provides the necessary data for inclusion in future meta-analyses to determine the strength of harms evidence in exercise oncology and to evaluate whether specific patient (e.g., age, cancer type, stage of disease at diagnosis) or exercise characteristics (e.g., frequency, intensity, mode, mode of delivery) influence harms.

We recognise that just as is the case for assessing potential exercise benefit, the goal for future ExHaRM iterations will be to reduce data collection burden (to participants, researchers, funding bodies etc) while maintaining comprehensive collection of meaningful information about exercise harms. Integrating a checklist of expected and clinically-important exercise-related harms (e.g., similar to the Common Terminology Criteria for Adverse Events[11] and the associated patient-reported version (PRO-CTCAE)[22]) is a proposed development for future iterations of ExHaRM. However, while this may prove useful in streamlining harms data collection, it would not replace the need to collect adverse outcomes through more time-consuming non-systematic approaches as promoted in the current ExHaRM version.

The identification of those adverse outcomes that are exercise-related is essential to the evaluation of the harms of exercise. The validity of the causality assessment process described in ExHaRM has not been formally evaluated and relies on experienced exercise and medical clinicians being involved, at a minimum, in the reviewing of causality attribution. Development of an algorithm similar to the causality probability scale developed by Naranjo et al[23] would be one approach to standardising causality attribution in exercise

For peer review only

oncology. However, in the interim, ExHaRM provides clear guidance to exercise professionals in judging the exercise-relatedness of adverse outcomes. We have found the involvement of a harms panel in ExHaRM extremely useful in reducing the potential for over- and under-reporting exercise-related adverse outcomes and ensuring consistency in the classification of adverse outcomes.

ExHaRM does not include a threshold at which exercise would be defined safe or unsafe. Decisions regarding the role of exercise in a specific context should be based on weighing evidence of the expected benefits against the potential harms. As is true with medical treatments, in some situations the benefits are sufficient that a higher level of concurrent harm is deemed acceptable, such as may be the case for life-saving chemotherapy. Within exercise oncology, some patient groups (e.g., those with advanced bone lesions or metastases) may be at higher risk of harm from exercise; however, if the benefits were of sufficient value to the population (e.g., maintained ability to independently complete activities of daily living) then exercise may be incorporated among care despite the harm profile.

With iterations of ExHaRM there was increasing recognition of the need to identify and consistently apply appropriate terms. Much discussion and deliberation has occurred over the use of all harms-related terms, but in particular the use of harms versus safety (which presupposes a positive outcome), and adverse outcomes versus adverse events. These discussions acknowledged that there are unintended negative consequences (i.e., harms) of changing lifestyle behaviours, that do not meet the definition of an adverse event as used in pharmacological harms reporting.[12] Adverse outcome (as used in the current iteration of ExHaRM) is a widely encompassing term which addresses the limitations of the term adverse event. There was also deliberation of whether adverse outcomes classified with the causality category of 'possible' should be defined, along with those specified as 'certain' and 'likely', as exercise-related adverse outcomes. This issue of causality categorisation also remains an area of debate in the harms reporting of pharmacological clinical trials.[24] Our current approach to include 'possible' is more inclusive, which was considered appropriate given the infancy of this specific area of harms reporting, but is a topic for future discussion and debate.

For peer review only

For peer review only - http://bmjopen.bmj.com/site/about/guidelines.xhtml

ExHaRM was developed for use in research. However, with minimal modifications this protocol could also be implemented in hospital-, university- and community-based exercise settings. Monitoring adverse outcomes (and near misses, in which no adverse outcome occurred but could have) within a clinic would facilitate risk mitigation through modification of processes (e.g., modification of prescription, type of equipment used, inclusion of contraindications or conditions that should be pre-screened), with the goal of reducing harms without compromising benefit. The collection of harms data in real-world settings also allows for the identification of rare adverse outcomes not previously identified during research, as well as those that may only occur outside of controlled research settings. Further, this harms reporting framework may also prove useful in exercise trials outside the oncological setting, as well as other lifestyle intervention trials beyond exercise.

Summary

There is a clear imperative to improve harms reporting in exercise oncology, and this imperative is heightened with the inclusion of understudied, rare cancers, and potentially higher-risk cohorts and interventions (e.g., participants with poorer prognoses and/or extensive comorbidities; higher intensity and/or less supervised exercise interventions) in exercise trials. In the short term, as per CONSORT guidelines,[10, 25] all exercise trials should have a harms reporting protocol. In the longer term, consensus regarding a standardised protocol and consistent use of harms-related terms, such as adverse outcomes, will facilitate rapid progress in this specific area of exercise oncology research.

For peer review only

Acknowledgements

The authors would like to acknowledge the contribution of participants, exercise professionals and chief investigators who were part of the SAFE, BRACE, ECHO and ECHO-R trials that were fundamental in the development of ExHaRM.

Contributors

RRS: Conceptualization, Methodology, Investigation, Data curation, Writing – Original Draft. CXS: Investigation, Data curation, Writing – Review & Editing. TMJ: Data curation, Writing – Review & Editing. NMM: Visualization, Writing – Review & Editing. RMD: Investigation, Writing – Review & Editing. SCH: Conceptualization, Supervision, Data curation, Writing – Review & Editing, Funding acquisition.

Funding

The suite of ihop exercise oncology trials that have contributed to the work presented have received funding from the following bodies: Icon Cancer Foundation (BRACE trial), Medical Research Future Fund (ECHO-R trial), and Cancer Australia, Cancer Council Australia, World Cancer Research Fund, Cancer Council Queensland and Griffith University (ECHO trial). CXS is supported by a Cancer Institute New South Wales Early Career Fellowships (2021/ECF1310).

Competing interests

The authors declare that they have no competing interests.

For peer review only - http://bmjopen.bmj.com/site/about/guidelines.xhtml

1. Hayes, S.C., et al., *Australian Association for Exercise and Sport Science position stand: optimising cancer outcomes through exercise.* J Sci Med Sport, 2009. **12**(4): p. 428-34.
2. Schmitz, K.H., et al., *American College of Sports Medicine roundtable on exercise guidelines for cancer survivors.* Med Sci Sports Exerc, 2010. **42**(7): p. 1409-26.
3. Campbell, K.L., et al., *Exercise Guidelines for Cancer Survivors: Consensus Statement from International Multidisciplinary Roundtable.* Med Sci Sports Exerc, 2019. **51**(11): p. 2375-2390.
4. Hayes, S.C., et al., *The Exercise and Sports Science Australia position statement: Exercise medicine in cancer management.* J Sci Med Sport, 2019. **22**(11): p. 1175-1199.
5. Reginster, J.Y., et al., *Importance of Safety in the Management of Osteoarthritis and the Need for Updated Meta-Analyses and Recommendations for Reporting of Harms.* Drugs Aging, 2019. **36**(Suppl 1): p. 3-6.
6. Adams, S.C., et al., *Comparing the reporting and conduct quality of exercise and pharmacological randomised controlled trials: a systematic review.* BMJ Open, 2021. **11**(8): p. e048218.
7. Niemeijer, A., et al., *Adverse events of exercise therapy in randomised controlled trials: a systematic review and meta-analysis.* Br J Sports Med, 2020. **54**(18): p. 1073-1080.
8. Singh, B., et al., *Exercise and colorectal cancer: a systematic review and meta-analysis of exercise safety, feasibility and effectiveness.* Int J Behav Nutr Phys Act, 2020. **17**(1): p. 122.
9. Singh, B., et al., *A systematic review and meta-analysis of the safety, feasibility and effect of exercise in women with stage II+ breast cancer.* Archives of Physical Medicine and Rehabilitation, 2018. https://doi.org/10.1016/j.apmr.2018.03.026.
10. Ioannidis, J.P., et al., *Better reporting of harms in randomized trials: an extension of the CONSORT statement.* Ann Intern Med, 2004. **141**(10): p. 781-8.
11. National Cancer Institute (U.S.), *Common terminology criteria for adverse events (CTCAE) - version 5.0*. NIH publication. 2017, Bethesda, Md.: U.S. Dept. of Health and Human Services, National Institutes of Health, National Cancer Institute.
12. Papaioannou, D., et al., *Adverse event recording failed to reflect potential harms: a review of trial protocols of behavioral, lifestyle and psychological therapy interventions.* J Clin Epidemiol, 2021. **136**: p. 64-76.
13. INTERNATIONAL COUNCIL FOR HARMONISATION OF TECHNICAL REQUIREMENTS FOR PHARMACEUTICALS FOR HUMAN USE (ICH), *Indexed ICH GCP guidelines with integrated addendum E6(R2), Step 4*. First edition. ed, ed. D.R. Hutchinson. 2016, Chobham, Surrey: International council for harmonisation. 64 pages.
14. National Institutes of Health (NIH), *NCI Guidelines For Investigators: ADVERSE EVENT REPORTING REQUIREMENTS*, U.S. Dept. of Health & Human Services, Editor. 2013.
15. National Health and Medical Research Council, *Guidance: Safety monitoring and reporting in clinical trials involving therapeutic goods*. 2016, National Health and Medical Research Council.: Canberra.
16. Spence, R., et al., *A Randomised, Comparative, Effectiveness Trial Evaluating Low- versus High-Level Supervision of an Exercise Intervention for Women with Breast Cancer: The SAFE Trial.* Cancers, 2022. **14**(6).

For peer review only - http://bmjopen.bmj.com/site/about/guidelines.xhtml

17. Sandler, C., et al., *Exercise during Chemotherapy for Recurrent Ovarian Cancer (ECHO-R): A Phase II trial evaluating feasibility, safety and efficacy of an individually-tailored exercise intervention during chemotherapy for recurrent ovarian cancer* Asia-Pacific Journal of Clinical Oncology, 2022. **Accepted**.
18. Gildea, G., Sandler CX., Elisadis, P., Pickersgill, D., Shevill, M., Bigg, V., Morrison, B., Bashford, J., Jonker, F., Hayes, S., *Evaluating exercise for patients with brain cancer – The BRACE study.* Asia-Pacific Journal of Clinical Oncology, 2021. **17**(S9).
19. Hayes S, F.M., Obermair A, Mileshkin L, Janda M, Gordon L, Barnes E, Beesley V, Eakin E, Sommeijer D, Martyn J, Stockler M, Gebski VAL, Naumann F, Schmitz K, Webb P, *Exercise during chemotherapy for ovarian cancer (ECHO): study design features and outcomes of a cancer Australia and cancer council australia funded randomised, controlled trial.* International journal of gynecological cancer, 2014. **24**: p. 200-201.
20. International Council for Harmonisation of Technical Requirements for Pharmaceuticals for Human Use, *MedDRA® Medical Dictionary for Regulatory Activities terminology. Retrieved from* https://www.meddra.org/. 2020.
21. Enrico, D., et al., *Analysis of adverse events attribution and reporting in cancer clinical trials: A systematic review.* Crit Rev Oncol Hematol, 2021. **160**: p. 103296.
22. Kluetz, P.G., et al., *Patient-Reported Outcomes in Cancer Clinical Trials: Measuring Symptomatic Adverse Events With the National Cancer Institute's Patient-Reported Outcomes Version of the Common Terminology Criteria for Adverse Events (PRO-CTCAE).* Am Soc Clin Oncol Educ Book, 2016. **35**: p. 67-73.
23. Naranjo, C.A., et al., *A method for estimating the probability of adverse drug reactions.* Clin Pharmacol Ther, 1981. **30**(2): p. 239-45.
24. Pande, S., *Causality or Relatedness Assessment in Adverse Drug Reaction and Its Relevance in Dermatology.* Indian J Dermatol, 2018. **63**(1): p. 18-21.
25. Schulz, K.F., et al., *CONSORT 2010 statement: updated guidelines for reporting parallel group randomised trials.* BMJ, 2010. **340**: p. c332.

For peer review only

Figure 1: An overview of the Exercise Harms Reporting Method (ExHaRM)
Note: See Box 1 (online supplemental material) for definitions and further explanation of terms used in this flow chart.

For peer review only

For peer review only - http://bmjopen.bmj.com/site/about/guidelines.xhtml

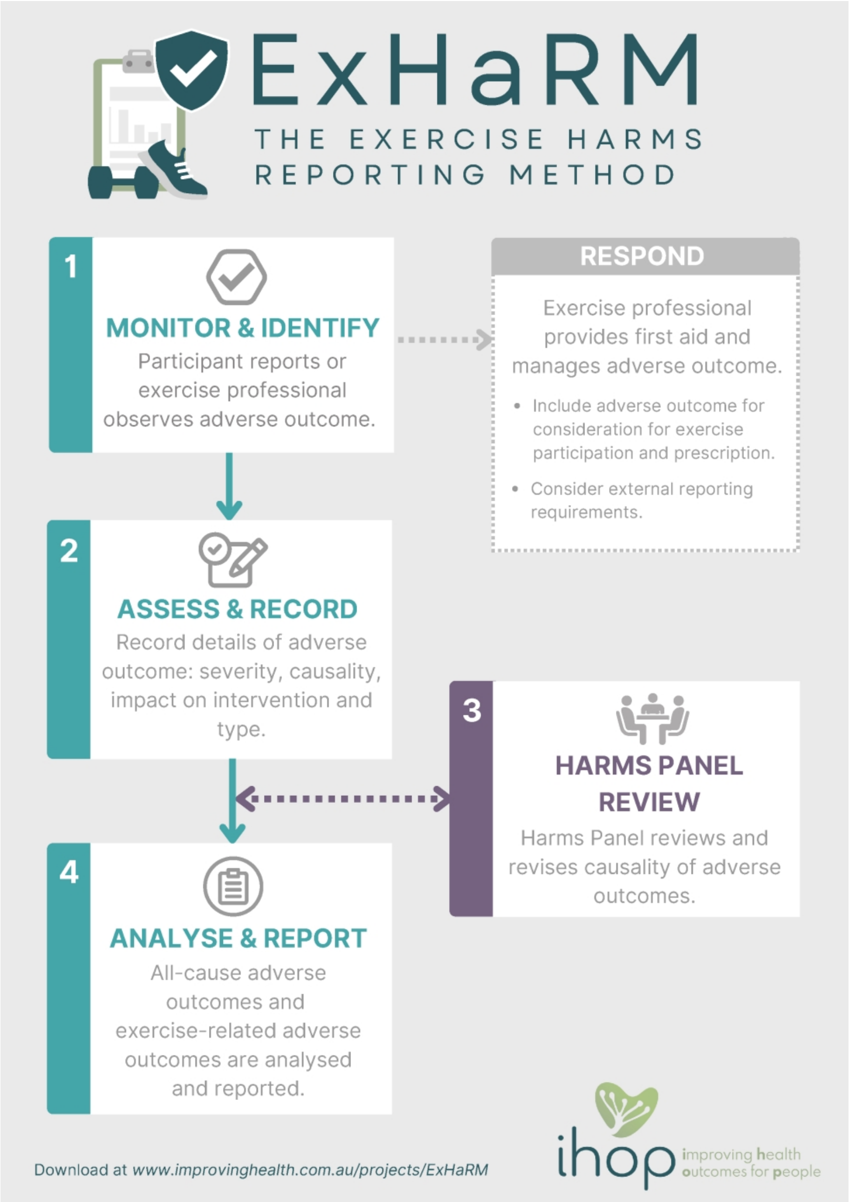

Figure 1: An overview of the Exercise Harms Reporting Method (ExHaRM)
Notes: See Table 1 for definitions and further explanation of terms used in this flow chart.

210x297mm (300 x 300 DPI)

Practical suggestions for harms reporting in exercise oncology: the Exercise Harms Reporting Method (ExHaRM)
Rosalind R Spence, Carolina X Sandler, Tamara L Jones, Nicole M McDonald, Riley M Dunn, Sandra C Hayes

Supplemental material - Box 1: Key terms and definitions used in the Exercise Harms Reporting Method (ExHaRM)

**Glossary of terms** (in alphabetical order)	
**Active surveillance of harms**	Participants are asked via structured questionnaires or interviews about the occurrence of *adverse outcomes*, or laboratory or other diagnostic tests which are performed at prespecified time intervals.<br>In the most basic form, this may involve asking the participant or exercise professional about the occurrence of "any undesirable occurrences" at set-time points during the study.
**Adverse event**	*Adverse event* is the most common measurement tool in harms reporting across disciplines.<br><br>*Adverse events* are any harmful or undesirable medical occurrence that occurs during participation in an intervention, irrespective of whether there is a causal relationship with the intervention.<br>A medical occurrence may include (but is not limited to):<br>• an acute injury;<br>• exacerbation of a chronic or past injury;<br>• new treatment-related side effect or symptom;<br>• increase in severity of treatment-related side effect or symptom;<br>• study withdrawal;<br>• early termination of treatment;<br>• disease progression or death from disease during intervention period.[1]
**Adverse Outcome**	An *adverse outcome* is any undesirable physical, psychological, social and economic outcome, experience or event. *Adverse outcomes* are all undesirable occurrences, irrespective of whether there was a causal relationship between the outcome and the intervention.
**Adverse Outcomes of interest**	*Adverse outcomes* that are important to the consumer (people living with and beyond cancer), clinicians (medical and exercise professional) and decision makers.<br>*Adverse outcomes of interest* are determined based on previous evidence of the harms of specific exercise (e.g., mode, intensity) in a given population (i.e., oncology and haematology). These often include: the most clinically serious adverse outcomes, common adverse outcomes, and adverse outcomes of importance to stakeholders and patients.<br>Examples of *adverse outcomes of interest*:

For peer review only

Practical suggestions for harms reporting in exercise oncology: the Exercise Harms Reporting Method (ExHaRM)

	• fracture rates in a population with pre-existing bone lesions (data collected via validated self-report questionnaires or extracted from medical records),<br>• newly diagnosed or exacerbated lymphoedema (this may be recorded by participants being prompted to report results of standard surveillance by their medical team or through study-specific testing, e.g., using bioelectrical impedance spectroscopy at pre-determined time points),<br>• issues with stomas or peripherally-inserted central catheters (data collected from medical records or prompted self-report at set time points).<br>For some *adverse outcomes of interest* it may be possible to collect data from the control and exercise groups, allowing comparison of risk.<br><br>Collecting *adverse outcomes of interest* should not exclude the active surveillance of other *adverse outcomes*, particularly given that what is important varies between individuals and situations.
**All-cause adverse outcomes**	*All-cause adverse outcomes* are all *adverse outcomes* identified in a trial, regardless of *causality* attribution.<br>*All-cause adverse outcomes* = non-exercise-related *adverse outcomes* + *exercise-related adverse outcomes.*
**Causality**	*Causality* describes the likelihood that an *adverse outcome* was caused by exercise, as judged by the exercise professional delivering the exercise intervention (or *Harms Panel*). *Causality* attribution is based on a range of factors, including the timing of the *adverse outcome* in relation to exercise versus other factors, the biological plausibility of the relationship and the expectedness of the *adverse outcome* (with regard to the nature, severity and frequency of the outcome) in the population being studied (i.e., is the adverse outcome an expected outcome of disease or treatment and therefore likely to occur even in the absence of exercise?).<br><br>**Categories:**<br>C = Certain: *adverse outcome* occurred within a plausible time relationship to completed exercise and cannot be explained by concurrent disease, treatment, or other drugs.<br><br>L = Likely: *adverse outcome* has a reasonable time sequence to completed exercise and unlikely to be attributed to concurrent disease, treatment, or other drugs. [sometimes termed “probable”, ExHaRM preferentially uses “likely” to reduce potential data errors due to confusing probable and possible]<br><br>P = Possible: *adverse outcome* has a reasonable time sequence to completed exercise but could also be explained by concurrent disease, treatment or other drugs.<br><br>U = Unlikely: *adverse outcome* has a temporal relationship to drug administration, treatment, or other medical event which makes a causal relationship with completed exercise improbable but not impossible, and other drugs or underlying disease provide a plausible explanation.

For peer review only - http://bmjopen.bmj.com/site/about/guidelines.xhtml

Practical suggestions for harms reporting in exercise oncology: the Exercise Harms Reporting Method (ExHaRM)

	NR = Not related: *adverse outcome* has no temporal relationship with exercise and/or *adverse outcome* has been attributed by other health professional to another cause (e.g., underlying disease or drug)<br><br>UC = Unclassified: This category should only be used when there is no way to categorise the *adverse outcome*. An exercise professional may use the term to identify an *adverse outcome* as requiring classification by the harms review panel, particularly when the exercise professional does not have appropriate scope or expertise to judge causality. A*dverse outcomes* recorded as unclassified by the exercise professional must be classified by the harms panel.<br><br>**Useful questions for exercise professional or Harms Panel to consider when attributing causality include:**<br>1. Is the *adverse outcome* a previously identified or a known response to exercise?<br>2. Has the *adverse outcome* occurred previously in this study (in this or another participant)?<br>3. Does the *adverse outcome* improve or stop when exercise is ceased?<br>4. Does the *adverse outcome* reoccur when exercise is re-commenced?<br>5. Is there a temporal relationship between the *adverse outcome* and exercise? Did exercise precede the *adverse outcome* and did the *adverse outcome* appear at an appropriate time interval after exercise (e.g., did the *adverse outcome* occur during exercise, or during a period post exercise that would mechanistically link the *adverse outcome* with the exercise. The post-exercise period may differ based on the specific *adverse outcome*. For example, a vasovagal episode is more likely to occur immediately post-exercise, whereas delayed-onset muscle soreness is more likely to occur 24-48 hours post-exercise)<br>6. Was the *adverse outcome* present at the beginning of the study (i.e., prior to commencing intervention / exercise)?<br>7. Can the participant's underlying clinical disease state (or comorbidities) explain the *adverse outcome*?<br>8. Are there any other potential causes for the *adverse outcome*?<br><br>(Questions adapted from "Oncology Clinical Trials: successful design, conduct and analysis",[2] and World Health Organisation–Uppsala Monitoring Centre Causality assessment system[3]).
**Common terminology criteria for adverse events (CTC-AE)**	The National Cancer Institute Common Terminology Criteria for Adverse Events (CTC-AE)[4] is a descriptive terminology which can be used for *harms* reporting.<br><br>A grading (severity) scale is provided for each adverse event term.<br><br>These criteria provide standardised language for describing the type and defining the severity of adverse events. The list of terms was developed to reflect *adverse events* within the context of medical treatment and therefore does not include a comprehensive list of terms relevant to exercise interventions. ExHarm uses the broad severity rating categories (i.e., grades 1 to 5) to grade the *severity* of *adverse outcomes* in the same way the CTC-AE grades *adverse events*, but primarily relies on MedDRA or other standardised language to describe the *adverse outcome*.
**Exercise-related adverse outcome**	An *adverse outcome* that is judged as having a reasonable causal relationship with participating in the exercise intervention.

For peer review only - http://bmjopen.bmj.com/site/about/guidelines.xhtml

Practical suggestions for harms reporting in exercise oncology: the Exercise Harms Reporting Method (ExHaRM)

	**Note:** 'reasonable causal relationship' means to convey, in general, that there is evidence or argument to suggest a causal relationship. "Reasonable causal relationship" includes the *causality* classifications of: "Possible", "Likely" and "Certain".<br><br>(Refer to *causality* term for full definitions of causality categories and recommended questions for determining attribution.)
**Harms panel**	The study-specific harms panel review the allocated causality of all *adverse outcomes*.<br><br>The composition of the panel will depend on the needs of the study. Most harms panels will include an exercise professional with substantial oncology experience, a medical oncologist, surgeon and/ or nurse, and senior research staff. It is preferable to include at least one member of the panel who has no other involvement in the study.
**Impact on intervention**	The impact of the *adverse outcome* describes how the participant's involvement in the intervention (i.e., exercise) is changed because of the *adverse outcome*.<br><br>Categories:<br>**M[1]:** modified exercise prescription (frequency, intensity, type, duration, volume)<br>**A[1]:** adjusted frequency of contact (e.g., increased or decreased frequency of contact with exercise professional),<br>**TI [2]:** temporary interruption from the intervention (contact and exercise),<br>**PW [2]:** permanent withdrawal from intervention (with or without withdrawal from the study),<br>**D:** Death<br><br>[1] Adjusted frequency of contact and modified exercise prescription may occur concurrently.<br>[2] Permanent withdrawal and temporary interruption may need to be coded or recoded retrospectively.
**Medical dictionary for Regulatory Activities (MedDRA®)[5]**	MedDRA® is the international medical terminology developed under the auspices of the International Council for Harmonisation of Technical Requirements for Pharmaceuticals for Human Use (ICH). It provides a standardised method to describe medical events.<br>Using standardised language allows for comparison between studies and aggregate reporting via meta-analyses.<br><br>MedDRA is searchable via a browser which is accessible via internet, mobile device or desktop (https://www.meddra.org/). Access requires a subscription which is free of charge for non-commercial / not for profit organisations.<br>MedDRA® trademark is registered by ICH.<br><br>A limitation of MedDRA is that it is medical terminology and does not capture some outcomes, such as abnormal responses to exercise. Study-specific language lists can be developed for use in instances that are not able to be described by MedDRA.

For peer review only - http://bmjopen.bmj.com/site/about/guidelines.xhtml

Practical suggestions for harms reporting in exercise oncology: the Exercise Harms Reporting Method (ExHaRM)

**Non-systematic surveillance of harms**	Collection of *adverse outcomes* that does not use a systematic approach (i.e., researchers do not ask about specific *adverse outcomes* or apply standardised testing). This approach usually involves asking participants to report the presence of *adverse outcomes* they have noticed (this can be through active or passive surveillance).
**Passive surveillance of harms**	Participants are not specifically asked about or tested for the occurrence of *adverse outcome*. Rather, *adverse outcomes* are identified based on participant or exercise professional reports made on their own initiative. This is also called spontaneous reporting.
**Persistent adverse outcome**	A *persistent adverse outcome* is an *adverse outcome* that extends continuously, without resolution.<br>The *adverse outcome* is reported only once unless the grade changes. If the grade becomes more or less severe the *adverse outcome* is reported again, as a new *adverse outcome* with the new grade.
**Recall period**	The period between each occasion of *adverse outcome* reporting (by participant or clinician).<br>E.g., If the exercise professional asks the participant to report any *adverse outcomes* that have occurred since the previous session that was 7 days ago, then the *recall period* would be 7 days.
**Recurrent adverse outcome**	A *recurrent adverse outcome* is an *adverse outcome* that occurs and resolves.<br>An *adverse outcome* that resolves and then recurs should be reported at each recurrence.
**Serious adverse event**	The term *Serious adverse event* has not been used in in ExHaRM. We acknowledge the term here as it is often a reporting requirement of ethics or institutional boards.<br><br>*Serious adverse events* are *adverse events* that meet a specific definition, separate to severity. The term is primarily used in defining regulatory reporting obligations for clinical trials. In ExHaRM *adverse events* are captured by the umbrella term *adverse outcome*. *Adverse outcomes* are divided by grade into mild-moderate (grade 1-2) and severe (≥ grade 3), rather than being defined as serious and non-serious.<br><br>*Serious adverse events*[6] are any exercise-related *adverse events* (i.e., "undesirable medical occurrences") occurring at any dose of an intervention that results in ANY of the following outcomes:<br>1) Death.<br>2) A life-threatening experience.<br>3) Inpatient hospitalization or prolongation of existing hospitalization (for >24 hours).<br>4) A persistent or significant incapacity or substantial disruption of the ability to conduct normal life functions<br>5) A congenital anomaly/birth defect.<br>6) Important Medical Events that may not result in death, be life threatening, or require hospitalization may be considered a serious adverse experience when, based upon medical judgment, they may jeopardize the patient or subject and may require medical or surgical intervention to prevent one of the outcomes listed in this definition

For peer review only - http://bmjopen.bmj.com/site/about/guidelines.xhtml

Practical suggestions for harms reporting in exercise oncology: the Exercise Harms Reporting Method (ExHaRM)

	Reporting requirements are usually determined by ethics or institutional boards and in most cases there are specific reporting requirements for *serious adverse events*. Given clinical and legal implications of these events, it is recommended that all exercise interventions and programs have a process requiring exercise professionals to report these *serious adverse events* to senior staff. Further, it is recommended that any *adverse outcomes* that meet the above definition should be reported to supervisors/ senior staff for review, regardless of whether the exercise professional judged the event to be exercise-related. It is recommended that *serious adverse events* (or *adverse outcomes* where *causality* could not be confidently assessed, but that otherwise met any serious adverse event criteria) are reported to the harms panel within 48 hours for immediate review.
**Severity / Grade**	*Adverse outcomes* are given a severity rating based on a grading scale of 1-5 (as per CTC-AE).<br><br>Severity may also be grouped into categories of mild-moderate (grade 1-2 *adverse outcomes*) or severe (grade 3-5 *adverse outcomes*).<br>Note: A severe *adverse outcome*, is NOT the same as *serious adverse event* which is defined separately (see "*serious adverse events*")
**Grade 1**	Mild; asymptomatic or mild symptoms; clinical or diagnostic observations only; medical treatment not indicated.<br><br>*ExHaRM application: "no/minimal disruption to activities of daily living"*
**Grade 2**	Moderate; minimal, local or noninvasive medical treatment indicated; limiting age-appropriate instrumental activities of daily living.<br>[Instrumental activities of daily living refer to activities such as preparing meals, shopping for groceries, using the telephone, managing money, etc.]<br><br>*ExHaRM application: "some disruption to instrumental activities of daily living"*
**Grade 3**	Severe or medically significant but not immediately life-threatening; hospitalization or prolongation of hospitalization indicated; disabling; substantially limiting self-care activities of daily living.<br>[Self-care activities of daily living refer to activities including bathing, dressing and undressing, feeding self, using the toilet, taking medications.]<br><br>*ExHaRM application: "complete disruption to self-care activities of daily living"*
**Grade 4**	Life-threatening consequences; urgent medical treatment indicated.
**Grade 5**	Death related to *adverse outcome*.
**Systematic surveillance of harms**	Asking every participant (at defined time points) about specific *adverse outcomes or* performing specific laboratory or other diagnostic tests. Systematic surveillance is, by definition, one approach to active surveillance of harms.

For peer review only

Practical suggestions for harms reporting in exercise oncology: the Exercise Harms Reporting Method (ExHaRM)

	Systematic methods, such as querying participants using a comprehensive checklist or standardized laboratory tests, are more likely than non-systematic methods to identify all occurrences of an *adverse outcome*.
**Type of adverse outcome**	Relevant categories of *Type of adverse outcome* may depend on the type of intervention and any *harms of interest* collected.<br><br>*Example list and reporting codes*<br>**Ex:** normal response to exercise (e.g., DOMS),<br>**AbEx:** abnormal response to exercise,<br>**SE:** expected disease- or treatment-related side effect,<br>**SE+:** exacerbation of disease- or treatment-related side effect,<br>**In:** injury,<br>**O:** other (describe)

For peer review only

References

1. INTERNATIONAL COUNCIL FOR HARMONISATION OF TECHNICAL REQUIREMENTS FOR PHARMACEUTICALS FOR HUMAN USE (ICH), *Indexed ICH GCP guidelines with integrated addendum E6(R2), Step 4*. First edition, ed. D.R. Hutchinson. 2016, Chobham, Surrey: International council for harmonisation.
2. Kelly, W. and S. Halabi, *Oncology Clinical Trials: Successful Design, Conduct, and Analysis*. 2nd ed. 2018.
3. World Health Organization (WHO)-Uppsala Monitoring Centre *The use of the WHO-UMC system for standardized case causality assessment*.
4. National Cancer Institute (U.S.), *Common terminology criteria for adverse events (CTCAE) - version 5.0*. NIH publication. 2017, Bethesda, Md.: U.S. Dept. of Health and Human Services, National Institutes of Health, National Cancer Institute.
5. International Council for Harmonisation of Technical Requirements for Pharmaceuticals for Human Use, *MedDRA® Medical Dictionary for Regulatory Activities terminology. Retrieved from https://www.meddra.org/*. 2020.
6. National Institutes of Health (NIH), *NCI Guidelines For Investigators: ADVERSE EVENT REPORTING REQUIREMENTS*, U.S. Dept. of Health & Human Services, Editor. 2013.
